# Transcription Factor KLF10 Constrains IL-17-Committed Vγ4^+^ γδ T Cells

**DOI:** 10.3389/fimmu.2018.00196

**Published:** 2018-02-28

**Authors:** Girak Kim, Min Jeong Gu, Soo Ji Kim, Kwang Hyun Ko, Yoon-Chul Kye, Cheol Gyun Kim, Jae-Ho Cho, Woon-Kyu Lee, Ki-Duk Song, Hyuk Chu, Yeong-Min Park, Seung Hyun Han, Cheol-Heui Yun

**Affiliations:** ^1^Department of Agricultural Biotechnology and Research Institute of Agriculture and Life Sciences, Seoul National University, Seoul, South Korea; ^2^Academy of Immunology and Microbiology, Institute for Basic Science, Pohang, South Korea; ^3^College of Medicine, Inha University, Incheon, South Korea; ^4^Department of Animal Biotechnology, Chonbuk National University, Jeonju, South Korea; ^5^Division of Bacterial Disease Research, Center for Infectious Disease Research, National Institute of Health, Korea Centers for Disease Control and Prevention, Osong, South Korea; ^6^Department of Immunology, Laboratory of Dendritic Cell Differentiation and Regulation, School of Medicine, Konkuk University, Chungju, South Korea; ^7^Department of Oral Microbiology and Immunology, DRI and BK21 Plus Program, School of Dentistry, Seoul National University, Seoul, South Korea; ^8^Center for Food Bioconvergence, Seoul National University, Seoul, South Korea; ^9^Institute of Green Bio Science Technology, Seoul National University, Pyeongchang, South Korea

**Keywords:** KLF10, γδ T cells, IL-17, homeostasis, Innate-like γδ-17

## Abstract

γδ T cells, known to be an important source of innate IL-17 in mice, provide critical contributions to host immune responses. Development and function of γδ T cells are directed by networks of diverse transcription factors (TFs). Here, we examine the role of the zinc finger TFs, Kruppel-like factor 10 (KLF10), in the regulation of IL-17-committed CD27^−^ γδ T (γδ^27−^-17) cells. We found selective augmentation of Vγ4^+^ γδ^27−^ cells with higher IL-17 production in KLF10-deficient mice. Surprisingly, KLF10-deficient CD127^hi^ Vγ4^+^ γδ^27−^-17 cells expressed higher levels of CD5 than their wild-type counterparts, with hyper-responsiveness to cytokine, but not T-cell receptor, stimuli. Thymic maturation of Vγ4^+^ γδ^27−^ cells was enhanced in newborn mice deficient in KLF10. Finally, a mixed bone marrow chimera study indicates that intrinsic KLF10 signaling is requisite to limit Vγ4^+^ γδ^27−^-17 cells. Collectively, these findings demonstrate that KLF10 regulates thymic development of Vγ4^+^ γδ^27−^ cells and their peripheral homeostasis at steady state.

## Introduction

Early studies on Kruppel-like factor 10 (KLF10), a transcription factor (TF) containing zinc finger DNA-binding domains, revealed its role in the induction of and balance between Foxp3^+^ regulatory T (Treg) cells and IL-17-producing T helper (Th17) cells (1–3). Stimulation of CD4^+^ T cells with T-cell receptor (TCR) or TGF-β transiently induces KLF10, which in turn suppresses TCR signaling or enhances TGF-β/Smad signaling, respectively. Therefore, KLF10-deficient CD4^+^ T cells that are hyper-activated by TCR stimuli are less differentiated into Treg cells than wild-type (WT) controls (1, 2), while Th17 cell differentiation is promoted (3). Nonetheless, the function of KLF10 *in vivo* is still unclear since the alteration of Treg cells in naïve KLF10-deficient mice is controversial (1–3) and the enrichment of Th17 cells in these mice has not been clearly reported. Most of all, the functions of KLF10 in other T lymphocytes producing IL-17, such as γδ T cells, are largely unknown.

At steady state, γδ T cells are only a minor subset of T lymphocytes but the major source of IL-17 (4–6). Innate-like IL-17-committed CD27^−^ γδ T (γδ^27−^-17) cells are present in peripheral lymph nodes (pLN) as well as regional tissues, including dermis, lung, and peritoneal cavity (5, 7, 8). Most peripheral γδ^27−^-17 cells stimulated by cytokines, for example, by IL-7 or by IL-1β plus IL-23, can be enriched in the absence of TCR activation (8, 9), and thus respond rapidly to infection or tissue dysregulation. Although TCR signaling is involved in γδ-lineage commitment and functional decision in the thymus (10–12), peripheral homeostasis and activity of γδ^27−^-17 cells is weakly dependent on TCR ligation, which triggers strong activation of γδ^27+^ cells (8, 13–16). γδ^27−^-17 cells mainly consist of Vγ4^+^ and Vγ6^+^ subsets (Tonegawa nomenclature) (17) and phenotypically resemble effector memory cells (CD44^hi^CD62L^lo^CD127^hi^) (5, 9), mostly expressing a unique marker, CCR6^+^NK1.1^−^ (18). It has been suggested that γδ^27−^-17 cells develop predominantly from early embryonic stage up to shortly after birth (19–21). However, whereas maturation of Vγ4^+^ γδ^27−^-17 cells occurs in the neonatal thymus (22), Vγ4^+^ γδ^27−^-17 cells can be still reconstituted by bone marrow (BM) cells (22, 23).

Thymic development of γδ T cells is regulated by discrete TCR strengths and TCR-independent signaling modalities, which involve exogenous stimuli (TGF-β and IL-7) and/or intrinsic pre-programming of a gene regulatory network of diverse TFs (24–26). It is plausible that a weak TCR strength is required for the development of innate-like γδ^27−^-17 cells and, thus, IL-17-producing capacity is considered to emerge by default from uncommitted early thymocytes (10, 11, 27). However, other reports argue that innate-like γδ-17 cells are dependent on strong TCR signals for their thymic development (13), leaving the role of TCR signaling in the generation of innate-like γδ^27−^-17 cells unclear. Moreover, TGF-βR or IL-7R signaling, as well as the TF Sox13, promote γδ^27−^-17 cell development through a TCR-independent signaling pathway (5, 9, 22); in particular, Sox13 selectively regulates Vγ4^+^ γδ^27−^-17 cell development (22).

Here, we identify KLF10 as a novel TF that negatively regulates the development and homeostasis of Vγ4^+^ γδ^27−^-17 cells. We found selective enlargement of IL-17-committed Vγ4^+^ γδ^27−^ cells, but not of other IL-17-producing αβ T cells, in KLF10-deficient mice. TCR or cytokine (IL-7 or IL-1β plus IL-23) stimulation on γδ T cells could induce KLF10, which in turn differently regulates γδ T-cell responsiveness to these stimuli. Moreover, KLF10 deficiency affected the expression level of CD5, a stable indicator of TCR strength, on mature Vγ4^+^ γδ^27−^-17 cells within the neonatal thymus. These results suggest that the biology of Vγ4^+^ γδ^27−^-17 cells is dependent on transcriptional control by KLF10, which is differentially associated with TCR and cytokine signaling.

## Materials and Methods

### Mice

KLF10-deficient mice with C57Bl/6 (B6) background were kindly provided by Dr. Woon Kyu Lee (Inha University, Incheon, South Korea) (28). B6.Rag1-deficient mice and B6.CD45.1 congenic mice were obtained from The Jackson Laboratory. All animals were bred and maintained under specific pathogen-free conditions at the Institute of Laboratory Animal Resource Seoul National University and treated in accordance with institutional guidelines that were approved by the Institutional Animal Care and Use Committee (SNU-140930-4-1).

### Cell Preparation

Mouse peripheral lymph nodes (cervical, axillary, brachial, and inguinal), mesenteric lymph node, spleen, thymus, and lung were homogenized by mechanical disaggregation, strained through a 70-µm strainer (BD Biosciences), and washed in RPMI 1640 medium containing 10% (vol/vol) fetal bovine serum (FBS). Peritoneal cells were obtained from peritoneal lavage in cold phosphate-buffered saline (PBS) containing 5% FBS.

### Flow Cytometry

Single-cell suspensions were first blocked with anti-CD16/32 antibody (93; eBioscience) and then stained with antibodies at 4°C for 20 min in staining buffer (1 × PBS containing 0.1% bovine serum albumin and 0.1% sodium azide). For intracellular cytokine staining, the cells were stimulated for 4 h with 50 ng/ml phorbol 12-myristate 13-acetate (PMA; Sigma-Aldrich) and 750 ng/ml ionomycin (Sigma-Aldrich) in the presence of brefeldin A (BD Biosciences). The cells were then fixed, permeabilized with a BD Cytofix/Cytoperm Kit according to the manufacturer’s instructions (BD Biosciences), and stained for IL-17 and IFN-γ. Intracellular phosphorylated protein staining was performed as described (29) with modifications. For assays of intracellular Ca^2+^ mobilization, cells from WT and knockout (KO) mice were first surface-stained with anti-CD45.2-PerCP-Cy5.5 and anti-CD45.2-Alexa 700, respectively, for subsequent identification of the two strains. Cells from both strains were mixed together at a ratio of 1:1 and loaded for 45 min at 37°C with the membrane-permeable fluorescent Ca^2+^ indicator dye, Indo-1 AM (Invitrogen), at a concentration of 4 µM in RPMI medium plus 5% (vol/vol) FBS. Cells were then stained for surface markers and were kept on ice. Before stimulation, cell aliquots were allowed to equilibrate to 37°C for 5 min and analyzed by flow cytometry. After acquisition of background intracellular Ca^2+^ concentrations for 30 s, cells were stimulated with biotinylated anti-CD3ε antibody and crosslinked by the addition of streptavidin. Samples were acquired on Canto II or Aria II (BD Biosciences) and data were analyzed with FlowJo software (TreeStar). Antibodies used for flow cytometric analyses were fluorochrome-labeled mAbs against mouse γδTCR (GL3), CD3ε (145-2C11), CD27 (LG.3A10 or LG.7F9), CD25 (PC61), CD69 (H1.2F3), CD5 (53-7.3), CD24 (M1/69), CD103 (2E7), CD122 (TM-b1), CD132 (TUGm2), CD127 (eBioSB/199), Ly6C (HK1.4), CD28 (E18), CD44 (IM7), CD62L (MEL-14), CCR6 (29-2L17), NK1.1 (PK136), CD45.1 (A20), CD45.2 (104), Vγ1 (2.11), Vγ4 (UC3-10A6), IL-17 (TC11-18H10), IFN-γ (XMG1.2), CD4 (RM4-5), CD8α (53-6.7), TCRβ (H57-597), pErk1/2 (D13.14.4E), pZap70 (n3kobu5), pSTAT3 (D3A7), and pSTAT5 (47/Stat5) (from BD Biosciences, Biolegend, eBioscience, or Cell Signaling Technology).

### Cell Sorting and Isolation

After blocking with anti-CD16/32, cells were negatively selected from pooled pLN and splenocytes using anti-TCRβ biotin, anti-CD45R biotin (RA3-6B2), and anti-CD11b (M1/70) antibodies together with MagniSort™ Streptavidin Negative Selection Beads according to the manufacturer’s instructions (eBioscience). The negatively selected cells were then stained with fluorochrome-labeled anti-γδTCR, anti-CD27, and/or anti-Vγ4 antibodies. γδ subsets (Vγ4^− γδ27+^, Vγ4+ γδ27+, Vγ4^− γδ27−^, and Vγ4^+ γδ27−^) were sorted using an Aria II with a purity of at least 98%. Alternatively, total γδ cells were manually isolated by negative selection as described previously (30) with modification. Biotin-conjugated mAbs against the following proteins were used: TCRβ, CD4, CD8α, CD45R, CD11b (M1/70), CD11c, Ly-6G (RB6-8C5), TER-119 (TER-119), and CD49b (DX5) (all from eBioscience).

### Cell Culture

Cells were cultured in RPMI 1640 containing 10% (vol/vol) FBS, 50 µM 2-mercaptoethanol, 1% (vol/vol) non-essential amino acids, 10 mM HEPES, and 1% (vol/vol) antibiotics/antimycotic solution (all from Invitrogen). For TCR stimulation, the cells were incubated for the indicated times with plate-bound anti-CD3ε mAb (0.1, 1, or 10 µg/ml; PeproTech) only or in combination with plate-bound anti-CD28 mAb (1 or 10 µg/ml; PeproTech). For cytokine stimulation, IL-7 (20 ng/ml; R&D Systems), TGF-β (10 ng/ml; R&D Systems), IL-6 (20 ng/ml; R&D Systems), IL-1β (10 ng/ml; PeproTech), and IL-23 (20 ng/ml; PeproTech) were added to the medium.

### Bone Marrow (BM) Chimeras

Bone marrow cells (5 × 10^6^ cells) from B6.CD45.2 wild-type (WT) or B6.CD45.2 KLF10-deficient mice were intravenously transferred to B6.CD45.1 mice, which were irradiated at 900 cGy. For mixed BM chimeras, B6.CD45.1/2 mice were lethally irradiated at 900 cGy and intravenously administered 5 × 10^6^ B6.CD45.1 WT cells mixed 1:1 with 5 × 10^6^ B6.CD45.2 KLF10-deficient BM cells. Recipient mice were sacrificed and analyzed for reconstituted γδ T cells after at least 12 weeks.

### Homeostatic Expansion

Single-cell suspensions of pLN cells obtained from B6.CD45.1 WT and B6.KLF10 knockout (KO) mice were stained with Cell Trace Violet (CVT; Invitrogen) and injected intravenously with 2 × 10^6^ cells into Rag-1-deficient mice. After 5 days, pLNs from the recipient mice were collected and examined for CTV dilution of the transferred T cells.

### RNA Extraction and Real-time qPCR

RNA was purified from cells using a Qiagen RNeasy kit. After reverse transcription into cDNA, PCR was performed with a StepOnePlus real-time PCR system (Applied Biosystems) and iTaq SYBR Green Supermix (Applied Biosystems) and relative expression was displayed in arbitrary units or as a percent of maximum expression, normalized to *Eef1a1* (encoding eukaryotic translation elongation factor 1α1; called “*Efa1*” hereafter) *via* the ΔΔCt method. The following primers were used: *Efa1* forward, 5′-TCCACCGAGCCACCATACA-3′, reverse, 5′-CCAACCAGAAATTGGCACAA-3′; *Klf10* forward, 5′-ACCCAGGGTGTGGCAAGAC-3′, reverse, 5′-AGCGAGCAAACCTCCTTTCA-3′; *Rorc* forward, 5′-TCAGCGCCCTGTGTTTTTCT-3′, reverse, 5′-CAAATTGTATTGCAGATGTTCCA-3′; *Sox13* forward, 5′-CTGCCACCTGGGTTACTTTGA-3′, reverse, 5′-GAGTGGCGTGATGAACATGTG-3′.

### Statistical Analyses

Prism software (GraphPad) was used for all statistical analyses. All quantitative data are shown as mean ±  standard deviation (SD) unless otherwise indicated. The two-tailed, paired *t*-test was used for BM chimeras. The two-tailed, unpaired *t*-test or two-way ANOVA followed by a Bonferroni *post hoc* test were used for all other data sets. Mean fluorescence intensity (MFI) indicates geometric MFI. Robust coefficient of variation (CV) defines 100 × 1/2 [intensity (at 84.13 percentile) − intensity (at 15.87 percentile)/Median] and was determined by FlowJo software. The robust CV is a normalized SD not as skewed by outlying values as the CV.

## Results

### KLF10 Controls Homeostatic Proliferation of γδ^27−^ Cells

**Figure 1:**
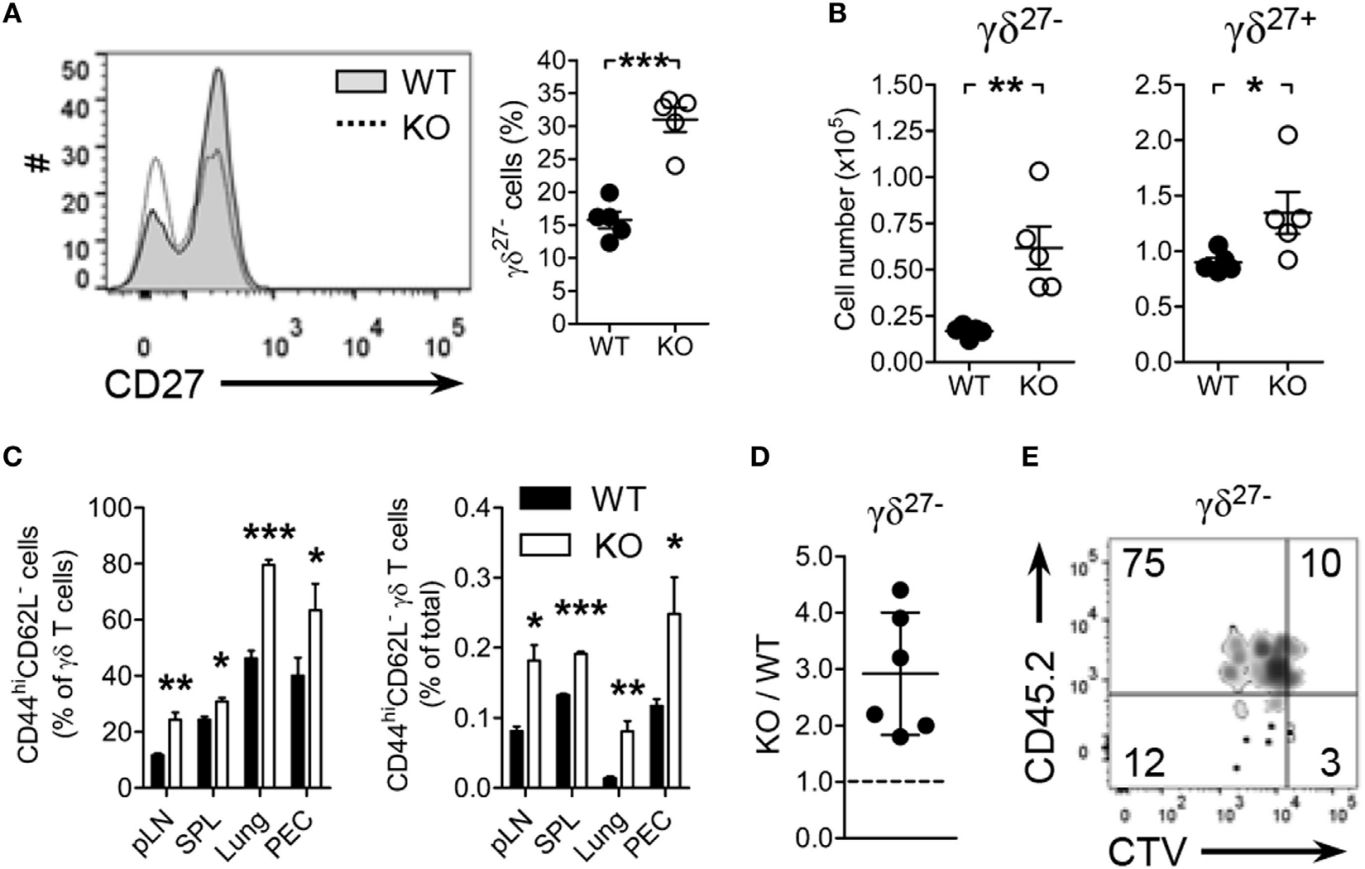
Kruppel-like factor 10 (KLF10) deficiency promotes peripheral γδ^27−^ cell expansion. **(A,B)** Overlayed histogram of CD27 expression on γδ T cells [**(A)**, left] and frequency of γδ^27−^ cells [**(A)**, right] and absolute number of γδ^27−^ and γδ^27+^ cells **(B)** in pooled peripheral lymph nodes (pLN; cervical, axillary, brachial, and inguinal) from wild-type (WT) and KLF10 knockout (KO) mice (*n* = 5 per group), as determined by flow cytometry. **(C)** Frequency of CD44^hi^CD62L^−^ cells among γδ T cells (left) and of CD44^hi^CD62L^−^ γδ T cells among total cells (right) in pLN, spleen (SPL), lung, and peritoneal exudate cells (PEC) from WT and KO mice (*n* = 5 per group), as determined by flow cytometry. Data are the mean ± SD. **(D,E)** The ratio of KO to WT γδ^27−^ cells **(D)**, homeostatic expansion of WT versus KO γδ^27−^ cells **(E)** from pLN cells of Rag-1-deficient mice, which had been co-injected with CTV-labeled CD45.1 WT and CD45.2 KO pLN cells at ratio of 2:1, were gated on CTV^+ γδ^TCR^+^CD27^−^ cells and then analyzed by flow cytometry after 5 days. Numbers in quadrants of the plot indicate percent of cells in each. Each symbol **(A,B)** represents an individual mouse; error bars are the mean ± SD. ns, non-significant; *P ≤ 0.05; **P ≤ 0.01; ***P ≤ 0.001. Data are representative of at least three **(A–C)** or two **(E)** independent experiments, or are pooled from two independent experiments **(D)**.

KLF10 has been reported to control Treg and Th17 cell induction (1, 3). Nonetheless, alterations of these cells in KLF10-deficient mice are still controversial (2, 3) and even the general T-cell status of the KO mice is not clearly defined (31). Therefore, we first examined the frequencies and absolute numbers of αβ and γδ T cells in the pLN, spleen, lung, and peritoneal cavity of KO mice under specific pathogen-free conditions. Compared with WT mice, levels of γδ T cells, but not conventional CD4^+^ and CD8^+^ αβ T cells, were significantly increased in pLN and lung of KO mice (Figures S1A,B in Supplementary Material). Analysis of discrete LNs (cervical, axillary, brachial, inguinal, and mesenteric; Figure S1C in Supplementary Material) confirmed the higher frequencies of γδ T cells, with the exception of mesenteric LN (mLN). Interestingly, the increase in γδ T cells was largely attributable to γδ^27−^ cell augmentation (Figures 1A,B). As expected, higher numbers of γδ^27−^ cells were observed in all LNs except for mLN (Figure S1D in Supplementary Material), in which innate-like γδ-17 cells are reported to be absent (5).

CD27 expression discriminated γδ subsets with CD44, CD62L, NK1.1, and CCR6; pLN γδ^27−^ cells were delineated as NK1.1^−^CCR6^+^CD44^hi^CD62L^−^ effector memory-phenotype cells (11, 32). Consistent with the augmentation of γδ^27−^ cells in KO mice, there were considerably more effector memory-phenotype (CD44^hi^CD62L^−^) γδ T cells in the analyzed organs (Figure 1C). By contrast, similar frequencies of effector memory-phenotype CD4^+^ T and CD8^+^ T cells in these organs were observed from both strains (Figure S2A in Supplementary Material). Intriguingly, the frequencies of Tregs (Figure S2B in Supplementary Material) and Th17 cells (Figure S2C in Supplementary Material, left column) among CD4^+^ T cells were unchanged in KO mice relative to those in WT mice. In summary, these data suggested that the generation of γδ^27−^ cells was enhanced in KO mice.

To explore the nature of γδ^27−^ cell augmentation in KO mice, we investigated whether KLF10 deficiency affected homeostatic proliferation of γδ^27−^ cells. Considering that the absolute number of KO pLN γδ^27−^ cells was about twice higher than that in WT pLN cells (Figure 1B), we transferred CD45.1 WT and CD45.2 KO pLN cells together into lymphopenic mice at 2:1 ratio; we confirmed that there were the same number of γδ^27−^ cells from both strains (data not shown). The homeostatic expansion of KO γδ^27−^ cells was superior to that of WT γδ^27−^ cells, with a ratio as great as threefold (Figures 1D,E). By contrast, we observed a similar expansion pattern of αβ T cells from both strains in recipient mice transferred with the pLN mixture at 1:1 ratio (Figures S2D–F in Supplementary Material). Therefore, these data suggested that KLF10 specifically inhibited γδ^27−^ cell expansion under lymphopenic conditions.

### KLF10 Deficiency Preferentially Expands Vγ4^+^ γδ^27−^-17 Cells

**Figure 2:**
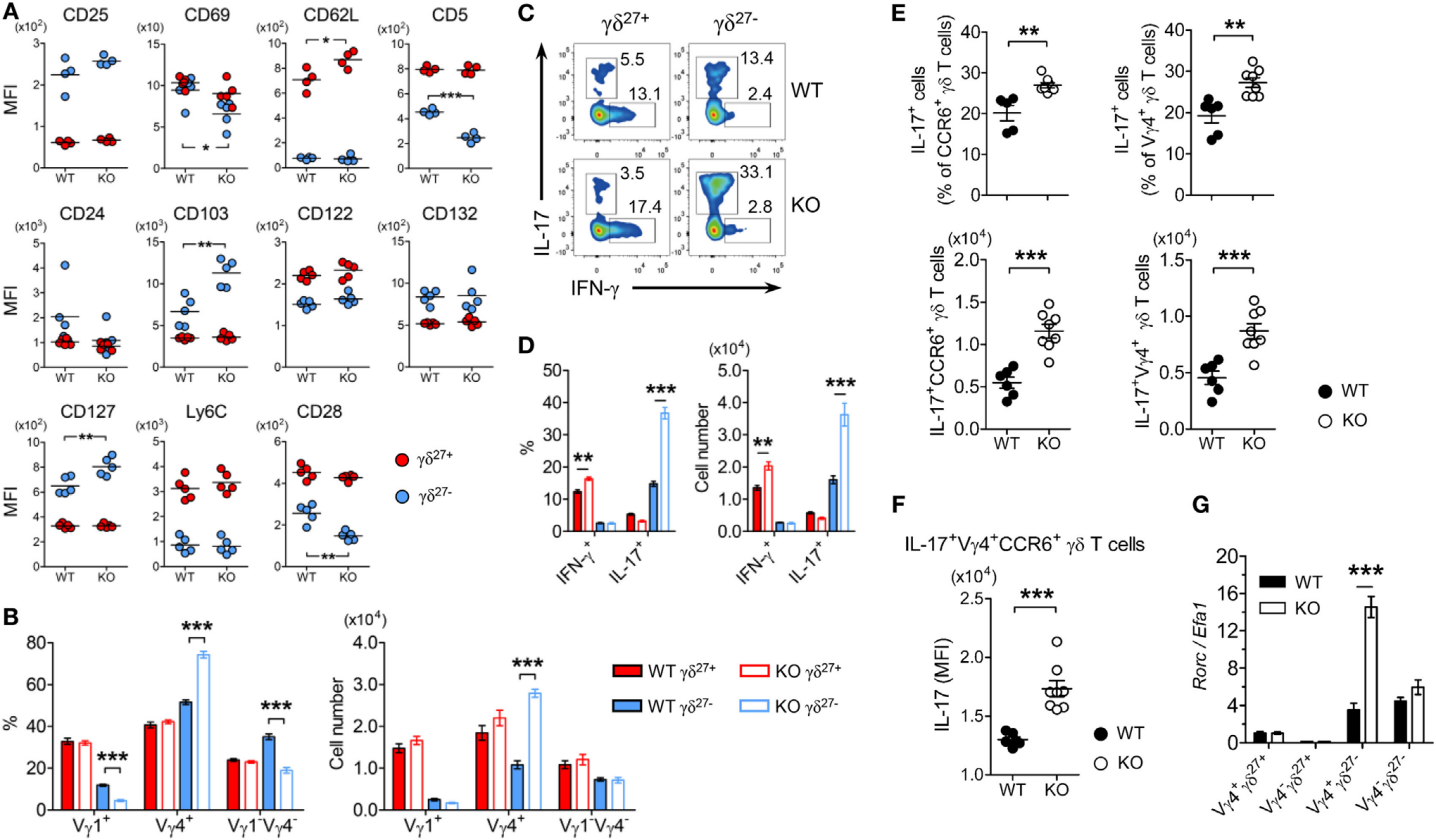
KLF10 deficiency selectively expands innate-like IL-17-committed Vγ4^+^ γδ^27−^ cells. **(A)** Mean fluorescence intensity (MFI) of each molecule expressed on γδ^27−^ (blue) and γδ^27+^ (red) cells in peripheral lymph nodes (pLN) from wild-type (WT) and knockout (KO) mice (*n* = 5 per group). **(B)** Frequency (left) and absolute number (right) of Vγ1^+^, Vγ4^+^, and Vγ1^−^Vγ4^−^ cells among γδ^27−^ and γδ^27+^ cells obtained as in **(A)**. **(C)** Intracellular IL-17 or IFN-γ expression in pooled pLN cells from WT and KO mice (*n* = 3 per group) after stimulation with phorbol 12-myristate 13-acetate (PMA) plus ionomycin, gated on γδ^27+^ or γδ^27−^ cells. Number adjacent to the gating indicates the percent of each population. **(D)** Frequency of IL-17^+^ or IFN-γ^+^ cells among γδ^27+^ and γδ^27−^ cells (left) and their absolute number (right) in WT and KO mice determined as in **(C)**. **(E)** Frequency of IL-17^+^ cells among CCR6^+^ (top, left) or Vγ4^+^ (top, right) γδ T cells and their absolute number (bottom) in pooled pLN cells from WT (*n* = 6) and KO mice (*n* = 8) after stimulation with PMA plus ionomycin. **(F)** IL-17 MFI of IL-17^+^Vγ4^+^CCR6^+^ γδ T cells determined as in **(E)**. **(G)** Real-time reverse transcription PCR analysis of *Rorc* expression in sorted Vγ4^+^ γδ^27+^, Vγ4^−^ γδ^27+^, Vγ4^+^ γδ^27−^, and Vγ4^−^ γδ^27−^ cells from pooled pLN and spleen of WT and KO mice (*n* = 10 per group), normalized to the housekeeping gene *Efa1*. Expression level of *Rorc* in WT Vγ4^+^ γδ^27+^ cells was set to 1. Data in **(B,D,G)** are mean ± SD. Each symbol **(A,E,F)** represents an individual mouse; error bars are the mean ± SD. *P ≤ 0.05; **P ≤ 0.01; ***P ≤ 0.001. Data are representative of at least three independent experiments **(A–F)** or two independent experiments **(G)**.

To determine phenotypic traits of γδ^27−^ cells expanded in a KLF10-deficient condition (Figures 1A–C), we investigated the expression of surface molecules involved in innate and adaptive immune features of γδ T cells. Interestingly, KO γδ^27−^ cells showed lower expression of CD69, CD28, and CD5, but higher expression of CD103 and CD127, than their WT counterparts (Figure 2A). To understand these changes in the light of Vγ chains, we examined the Vγ usage and found a Vγ4-biased composition of KO γδ^27−^ cells (Figure 2B, left). Indeed, Vγ4^+^ γδ^27−^ cells expressed lower CD5 and CD28 but higher CD127 and CD103 than Vγ4^−^ γδ^27−^ cells (data not shown), which demonstrated that the change in these surface molecules on γδ^27−^ cells (Figure 2A) could be attributed to the preferential distribution of a Vγ4 subset. In addition, only Vγ4^+^ γδ^27−^ cells were considerably enhanced in pLN, whereas the abundance of Vγ1^+^ γδ^27−^ and Vγ1^−^Vγ4^−^ γδ^27−^ cells was unchanged in KO mice compared with that in WT mice (Figure 2B, right). Collectively, our results indicated that Vγ4^+^ γδ^27−^ cells were selectively enriched under KLF10 deficiency.

As γδ^27−^ cells are innate-like IL-17-producing γδ T cells (γδ-17) (6), we examined intracellular levels of IL-17A. Consistent with the greater abundance of Vγ4^+^ γδ^27−^ (Figure 2B) and Vγ4^+^CCR6^+^ cells (data not shown) in pLN of KO mice, considerably more IL-17^+^ cells were observed in γδ^27−^ cells from KO pLN cells after stimulation with PMA plus ionomycin (Figures 2C,D). This was further confirmed by the increased number of IL-17^+^CCR6^+^ or IL-17^+^Vγ4^+^ γδ T cells (Figure 2E). Of note, IL-17 production by IL-17^+^Vγ4^+^CCR6^+^ γδ T cells was higher in the KLF10-deficient condition than in the normal condition, as measured by the MFI of intracellular IL-17 (Figure 2F), indicating that KLF10 constrained IL-17 production by Vγ4^+^ γδ^27−^ cells. Finally, KO Vγ4^+^ γδ^27−^ cells contained considerably higher levels of *Rorc* than their WT counterparts (Figure 2G). These results collectively suggested that KLF10 impaired the size of the innate-like Vγ4^+^ γδ^27−^-17 population together with their IL-17-producing capacity.

### KLF10 Differently Regulates γδ^27−^ Cell Responsiveness to Cytokine and TCR Stimuli

**Figure 3:**
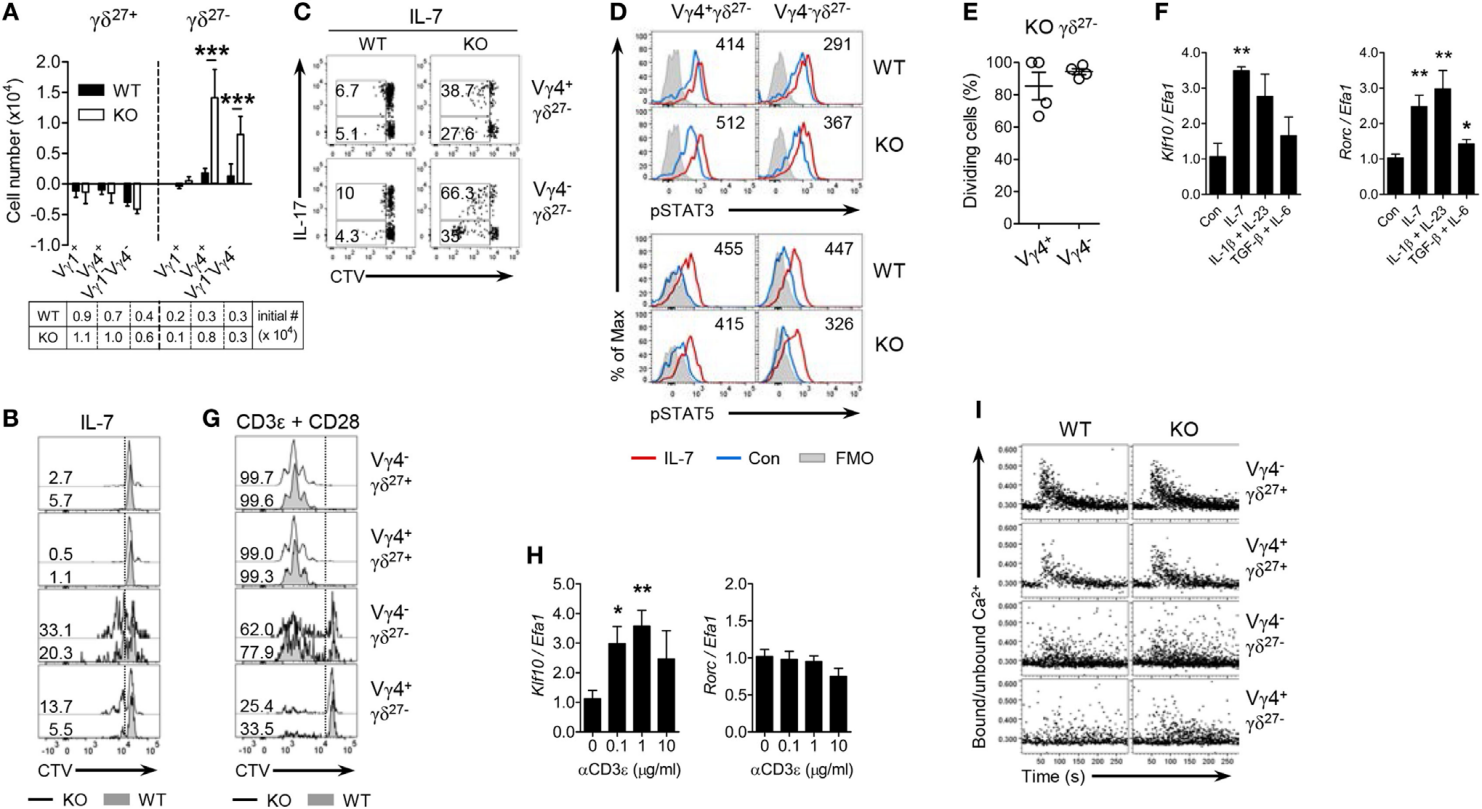
KLF10 inhibits γδ^27−^ cell responsiveness to cytokine but not TCR stimuli. **(A)** The change in numbers of Vγ1^+^, Vγ4^+^, and Vγ1^−^Vγ4^−^ of γδ^27+^ or γδ^27−^ cells from wild-type (WT) or knockout (KO) peripheral lymph node (pLN) cells (*n* = 3 per group) cultured with IL-7 (20 ng/ml) for 5 days, as determined by flow cytometry. The cell number at start was 5 × 10^6^. **(B,C)** Half-offset histogram of proliferation **(B)** and intracellular IL-17 expression [**(C)**; after stimulation with PMA plus ionomycin] in γδ T cells sorted from pooled pLN and spleen of WT and KO mouse (*n* = 10 per group), CTV-labeled and cultured with IL-7 (20 ng/ml) for 5 days. **(D)** Overlayed histogram of phosphorylated STAT3 or STAT5 in Vγ4^+^ γδ^27−^ or Vγ4^−^ γδ^27−^ cells from pooled WT or KO pLN cells (*n* ≥ 3 per group) cultured with IL-7 (20 ng/ml) for 30 min and assessed by flow cytometry. Numbers indicate mean fluorescence intensity variation between cells cultured with or without IL-7. Con, control; FMO, fluorescence minus one. **(E)** Percent of dividing cells among Vγ4^+^ γδ^27−^ or Vγ4^−^ γδ^27−^ cells from CD45.2 KO pLN cells, injected into Rag-1-deficient mouse and then analyzed by flow cytometry after 5 days as in Figure 1D, gated on CD45.2^+^CD27^−^CTV^+ γδ^TCR^+^ cells. **(F)** Real-time reverse transcription PCR analysis of *Klf10* and *Rorc* expression (normalized to the housekeeping gene *Efa1*) in sorted γδ^27−^ cells from pooled pLN and spleen of WT mice (*n* ≥ 10), cultured with or without IL-7 (20 ng/ml), IL-1β (10 ng/ml) plus IL-23 (20 ng/ml), or TGF-β (10 ng/ml) plus IL-6 (20 ng/ml) for 17 h. Expression levels of *Klf10* and *Rorc* in control (Con) were set to 1. **(G)** Half-offset histogram of proliferation of γδ T cells sorted as in **(B)**, CTV-labeled and cultured on plated-bound anti-CD3ε (0.1 µg/ml) and anti-CD28 (10 µg/ml) for 3 days. **(H)** Real-time reverse transcription PCR analysis of *Klf10* and *Rorc* expression (normalized to the housekeeping gene *Efa1)* in γδ T cells sorted as in **(F)**, cultured on plated-bound anti-CD3ε (αCD3ε; 0, 0.1, 1, and 10 µg/ml) for 3 h. Expression levels of *Klf10* and *Rorc* in cells without anti-CD3ε mAb were set to 1. **(I)** Intracellular Ca^2+^ mobilization in pLN cells obtained from WT and KO mice (*n* = 4), stained for surface markers to identify the indicated γδ subsets, stimulated *via* the TCR with biotinylated anti-CD3ε (20 µg/ml) followed by crosslinkage of the TCR with streptavidin (40 µg/ml) and then assayed over 5 min. Numbers in **(B,C,G)** indicate the percent of dividing cells **(B,G)** or of the gated population among IL-17 positive or negative cells in each panel **(C)**. Data in **(A,F,H)** are mean ± SD. Each symbol **(E)** represents an individual mouse; error bars are the mean ± SD ns, non-significant; *P ≤ 0.05; **P ≤ 0.01; ***P ≤ 0.001. Data are representative of at least three independent experiments **(A–C,E,I)** or two independent experiments **(D,F,H)**.

Peripheral homeostasis and IL-17 production of innate-like γδ-17 cells are mainly controlled by innate signaling triggered by cytokines, such as IL-7 and IL-1β plus IL-23 (8, 9). Thus, we determined whether KLF10 was involved in cytokine-signaling on Vγ4^+^ γδ^27−^ cells. Under IL-7 treatment the absolute number of Vγ4^+^ and Vγ1^−^Vγ4^−^ γδ^27−^ cells from KO mice was considerably increased compared with their WT counterparts (Figure 3A). Both γδ^27−^ subsets of KO mice exhibited greater proliferation (Figure 3B) and there were higher frequencies of IL-17^+^ expanding cells among KO γδ^27−^ subsets (Figure 3C). Because IL-7 enriches γδ^27−^-17 cells by activating STAT3 rather than STAT5 (9), we assessed STAT phosphorylation triggered by IL-7. IL-7 substantially activated STAT5 in both Vγ4^+^ and Vγ4^−^ γδ^27−^ cells from WT mice, whereas the phosphorylation of STAT3 was slightly induced by IL-7 (Figure 3D), consistent with the previous report showing the capacity of IL-7 to activate STAT3 in γδ^27−^ cells (9). Of note, compared to the normal condition, the KLF10 deficiency increased the level of phospho-STAT3 (pSTAT3), but not of pSTAT5, under IL-7 treatment (Figure 3D). Meanwhile, we observed the pSTAT3 induction in Vγ4^−^ γδ^27−^ cells when treated with IL-6, but not in Vγ4^+^ γδ^27−^ cells (Figure S3A in Supplementary Material). These data suggested that STAT3 activation might be involved in the hyper-responsiveness of KO γδ^27−^ cells to IL-7. On the other hand, contrary to the aforementioned *in vivo* Vγ4^+^ γδ^27−^-17 cell-specific regulation of KLF10 (Figure 2), *in vitro* hyper-responsiveness to IL-7 was observed in KO γδ^27−^ cells regardless of Vγ4 (Figures 3A–C). In addition, KLF10 deficiency promoted γδ^27−^ cells, irrespective of Vγ4, providing an advantage for homeostatic expansion in lymphopenic conditions (Figure 3E). Collectively, these results suggest a critical role for KLF10 in IL-7 signaling-mediated homeostasis of innate-like γδ^27−^-17 cells.

We next examined whether KLF10 is also involved in the reactivity of γδ^27−^ cells to inflammatory conditions such as IL-1β plus IL-23 that are known to induce IL-17 production by γδ T cells (8). Consistent with the results of IL-7 stimulation, γδ^27−^ cells from KO mice were hyper-responsive to IL-1β plus IL-23 (Figures S3B,C in Supplementary Material), which indicated that KLF10 also inhibited the activation of γδ^27−^ cells triggered by inflammatory stimuli. Of note, *Klf10* expression was increased in γδ^27−^ cells stimulated with IL-1β plus IL-23, as well as those stimulated with IL-7 (Figure 3F). On the other hand, KLF10 deficiency did not lead to an alteration in the calcium fluxes directly triggered by stimulation with PMA plus ionomycin, ruling out the possibility that the hyper-responsiveness of KO γδ^27−^ cells might be qualitatively unspecific to cytokines (Figure S3D in Supplementary Material). Together, these data suggested that homeostatic and inflammatory cytokine signaling could induce KLF10, which in turn, as a negative feedback signaling factor, might impair γδ^27−^ cell responsiveness to these innate stimuli.

Next, we sought to determine whether KLF10 might be involved in TCR-triggered activation of peripheral γδ T cells (13, 32). γδ^27+^ cells could readily expand under TCR/CD28 stimuli, whereas γδ^27−^ cells showed different patterns of proliferation and, especially, Vγ4^+^ γδ^27−^ cells hardly expanded (Figure 3G), supporting a preceding report that innate-like γδ^27−^ cells display hypo-responsive TCR signaling (13). Although *Klf10* could be significantly induced by TCR activation in total γδ T cells, in which γδ^27+^ cells accounted for up to 85% of the cells (Figure 3H), there were similar expansions of each γδ subset between WT and KO mice (Figure 3G). As previously reported (13), a rapid and transient increase in cytosolic calcium concentration triggered by TCR engagement was readily detected in γδ^27+^ cells, but not in γδ^27−^ cells; however, there were no differences in calcium fluxes between the strains (Figure 3I). Most of all, a Vγ4^+^ γδ^27−^ subset almost completely failed to phosphorylate ERK after TCR stimulation (Figure S3E in Supplementary Material). Therefore, KLF10 has a minor role in TCR-triggered activation of peripheral γδ subsets. To further investigate whether the level of engagement by the antigen or costimulatory receptor influenced KLF10 involvement in the TCR response, we treated the cells with different doses of agonist antibodies. Vγ4^+^ γδ^27−^ cells from both strains responded similarly to a high level of antigen engagement even in the absence of costimulatory signaling, but still showed an impaired proliferative response compared to that of γδ^27+^ cells (Figure S3F in Supplementary Material); similar results were found with costimulatory stimuli. Therefore, neither the strong antigen receptor nor costimulatory signaling caused a substantial discrepancy in TCR response of peripheral γδ subsets between the two strains.

### CD5^lo^CD127^hi^ γδ^27−^ Subsets As Innate-Like γδ-17 Cells

**Figure 4:**
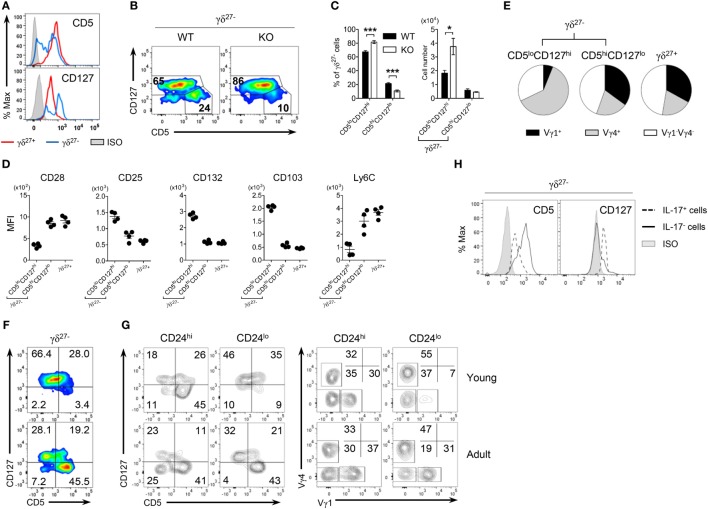
CD5^lo^CD127^hi^ γδ^27−^ cells are IL-17-competent γδ T cells. **(A)** Overlayed histogram of CD5 and CD127 expression on γδ^27+^ versus γδ^27−^ cells in peripheral lymph node (pLN) cells pooled from wild-type (WT) mice (*n* ≥ 4), as determined by flow cytometry. ISO, isotype control. **(B)** Pseudocolor plot of CD5 versus CD127 expression in pLN γδ^27−^ cells from WT and knockout (KO) mice (*n* ≥ 4 per group). **(C)** Percent of CD5^lo^CD127^hi^ and CD5^hi^CD127^lo^ cells [gated as in **(B)**] among γδ^27−^ cells (left) and their absolute numbers (right) in pLN cells from WT and KO mice (*n* ≥ 4 per group). **(D,E)** MFI of surface CD28, CD25, CD132, CD103, and Ly6C expression **(D)** and Vγ distribution **(E)** on CD5^lo^CD127^hi^ γδ^27−^, CD5^hi^CD127^lo^ γδ^27−^ [gated as in **(B)**] and γδ^27+^ cells obtained as in **(A)** (*n* = 4). **(F,G)** Pseudocolor plot of CD5 and CD127 on γδ^27−^ cells **(F)** and density plot of CD5 and CD127 [**(G)**, left] or Vγ1 and Vγ4 [**(G)**, right] on CD24^hi^ or CD24^lo^ γδ^27−^ cells in pLN cells from 2-week-old (young) and 8-week-old (adult) WT mice (*n* ≥ 4 per each). **(H)** Overlayed histogram of CD5 and CD127 on IL-17^+^ or IL-17^−^ γδ^27−^ cells obtained as in **(A)** (*n* = 3), after stimulation with PMA plus ionomycin. Numbers in outlined areas or quadrants of plots indicate percent of cells in each. Data in **(C)** are mean ± SD. Each symbol **(D)** represents an individual mouse; error bars are the mean ± SD. *P ≤ 0.05; ***P ≤ 0.001. Data are representative of at least three independent experiments.

The expression level of CD5, a stable indicator of TCR strength, was lower in γδ^27−^ cells than in γδ^27+^ cells (Figure 2A), indicating that γδ^27−^ cells might receive a relatively weak TCR strength compared with γδ^27+^ cells; this is in line with the fact that a weak TCR-signal strength is required for thymic development of innate-like γδ-17 cells (11, 33). Intriguingly, when we monitored CD5 expression (Figure 4A), γδ^27−^ cells contained two different populations (CD5^high^ and CD5^low^), meaning that they are a heterogeneous group receiving discrete TCR-signal strengths. Such a two-peak pattern was also observed for CD127 (IL-7 receptor-α, IL-7Rα), one of the markers identifying innate-like γδ-17 cells (Figure 4A) (9, 13), allowing us to distinguish CD5^lo^CD127^hi^ and CD5^hi^CD127^lo^ cells (Figure 4B) and further presume that CD5^lo^CD127^hi^ γδ^27−^ cells might represent the innate-like γδ-17 cells. Indeed, the CD5^lo^CD127^hi^ γδ^27−^ subset was greater in KO mice, whereas the CD5^hi^CD127^lo^ γδ^27−^ subset was present in normal numbers (Figure 4C).

Interestingly, CD5^hi^CD127^lo^ γδ^27−^ cells closely resembled γδ^27+^ cells rather than CD5^lo^CD127^hi^ γδ^27−^ cells in terms of surface protein expression (Figure 4D) and Vγ usage (Figure 4E). We noted that the frequency of CD5^hi^CD127^lo^ cells among γδ^27−^ cells was very low (3.4%) in young mice (2 weeks old) but greatly increased (45.5%) in the adult (8 weeks old) (Figure 4F). These cells mainly appeared within mature (CD24^lo^) γδ^27−^ cells of the thymus (Figure S4 in Supplementary Material) and pLN (Figure 4G), and were accompanied by higher levels of Vγ1^+^ cells. In addition, CD5^hi^Vγ1^+^ cells emerged among immature (CD24^hi^) γδ^27−^ cells of the adult thymus (Figure S4 in Supplementary Material), in accordance with the previous report on sequential Vγ waves with age (34) and possibly indicating that CD5^hi^CD127^lo^ γδ^27−^ cells might be generated in the adult rather than the fetal/neonatal thymus.

In contrast to CD5^hi^CD127^lo^ γδ^27−^ and γδ^27+^ cells, CD5^lo^CD127^hi^ γδ^27−^ cells had common γ chain receptor signal-dependent and epithelial-homing phenotypes with Vγ4^+^ subset dominance (Figures 4D,E). In particular, IL-17^+^ γδ^27−^ cells were CD5^lo^CD127^hi^ (Figure 4H), which ultimately validated CD5^lo^CD127^hi^ γδ^27−^ cells as “virtually” innate-like γδ-17 cells.

### CD5^int^Vγ4^+^CD127^hi^ γδ^27−^-17 Cell Development in KLF10-Deficient Mice

**Figure 5:**
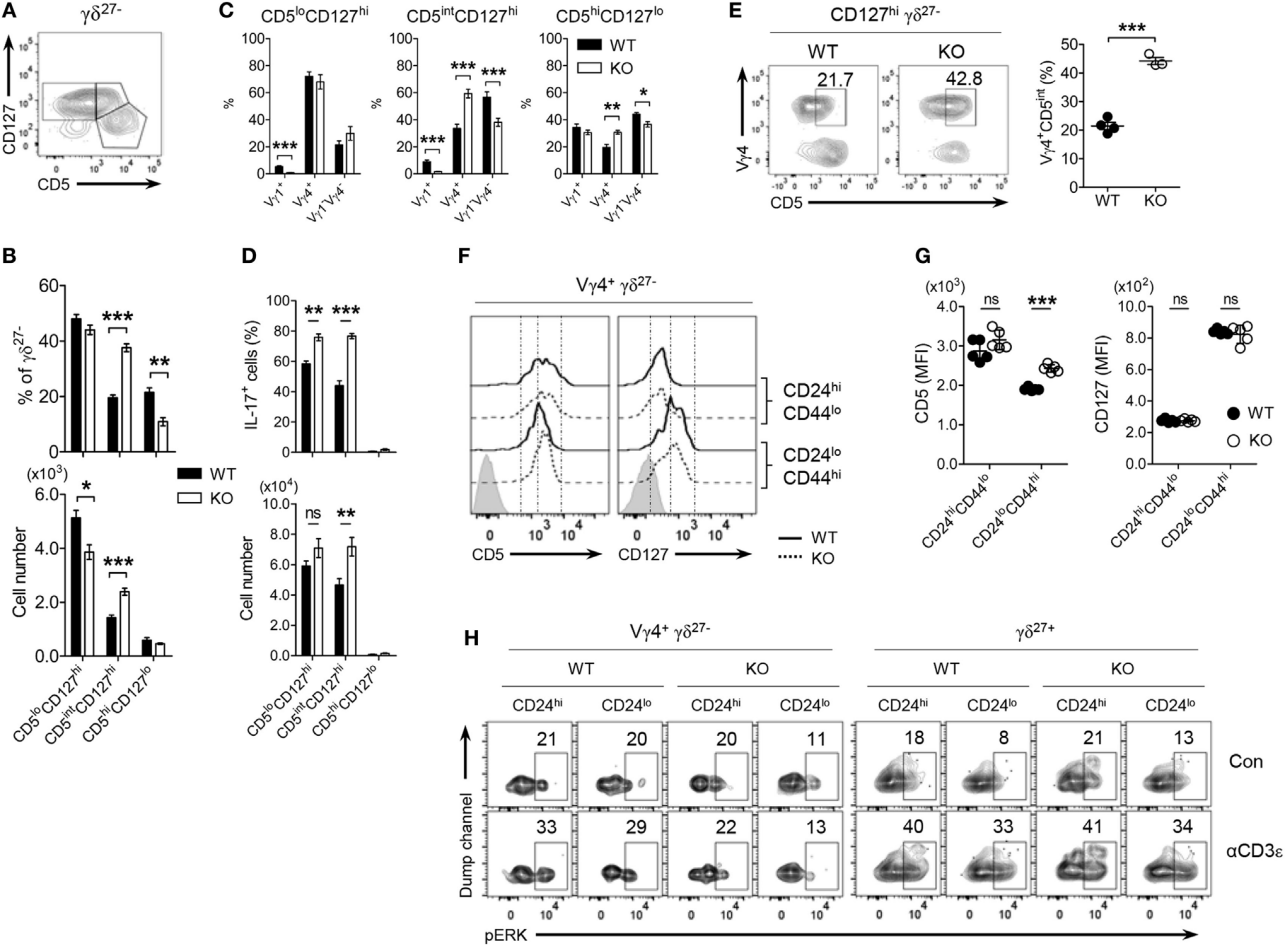
Vγ4^+^ γδ^27−^ cells developmentally express relatively higher CD5 under KLF10-deficient condition. **(A)** Density plot of CD5 and CD127 on peripheral lymph node (pLN) γδ^27−^ cells from wild-type (WT) mice (*n* ≥ 4). **(B–D)** Percent of CD5^lo^CD127^hi^, CD5^int^CD127^hi^ and CD5^hi^CD127^lo^ [gated as in **(A)**] among γδ^27−^ cells [**(B)**, top], their absolute numbers [**(B)**, bottom] and Vγ distribution **(C)**, and frequency of IL-17^+^ cells among the each gated group [**(D)**, top; after stimulation with phorbol myristate acetate plus ionomycin] and their absolute numbers [**(D)**, bottom] in pLN cells from WT and knockout (KO) mice (*n* ≥ 5 per group). **(E)** Density plot of CD5 and Vγ4 on CD127^hi^ γδ^27−^ cells (left) and percent of Vγ4^+^CD5^int^ cells among them (right) in pLN cells from WT and KO mice (*n* = 4). **(F,G)** Half-offset histogram of CD5 and CD127 **(F)** and their mean fluorescence intensity (MFI) **(G)** on CD24^hi^ or CD24^lo^ Vγ4^+^ γδ^27−^ cells in thymocytes from neonatal (day 4 after birth) WT and KO mice (*n* = 5 per group). **(H)** Density plot of phosphorylated ERK in CD24^hi^ or CD24^lo^ cells of Vγ4^+^ γδ^27−^ and γδ^27+^ cells from neonatal thymocytes obtained as in **(F)**, stimulated with soluble anti-CD3ε (1 µg/ml) for 3 min. Con, control. Numbers adjacent outlined areas of plots **(E,H)** indicate percent of cells in each. Data in **(B–D)** are mean ± SD. Each symbol **(E,G)** represents an individual mouse; error bars are the mean ± SD ns, non-significant; *P ≤ 0.05; **P ≤ 0.01; ***P ≤ 0.001. Data are representative of at least three independent experiments **(A–E)** or two independent experiments **(F–H)**.

By scrutinizing the expression patterns of CD5 and CD127 on γδ^27−^ cells, depicted in a pseudocolor plot (Figure 4B), we could recognize another peak of CD5 that was relatively high in CD5^lo^CD127^hi^ γδ^27−^ cells under KLF10 deficiency, but otherwise was barely detectable under normal conditions. Thus, we further distinguished CD5^int^ cells from CD5^lo^CD127^hi^ γδ^27−^ cells (Figure 5A) and found that the CD5^int^CD127^hi^ subgroup was expanded in a KLF10-deficient condition (Figure 5B). This enriched sub-group skewed toward a Vγ4 chain (Figure 5C), consistent with the selective enrichment of a Vγ4^+^ γδ^27−^ subset in KO mice (Figure 2B). Indeed, IL-17 production was observed in both CD5^lo^ and CD5^int^ CD127^hi^ γδ^27−^ cells, and the latter population (IL-17^+^CD5^int^CD127^lo^) was genuinely augmented by KLF10 deletion (Figure 5D). Taken together, these findings indicated that KLF10 deficiency selectively expanded innate-like IL-17-competent CD5^int^Vγ4^+^CD127^hi^ γδ^27−^ cells.

In normal conditions, CD5^lo^ and CD5^int^ CD127^hi^ γδ^27−^ subgroups exhibited preferential distribution of Vγ4^+^ and Vγ1^−^Vγ4^−^ subsets, respectively (Figure 5C), raising the possibility that KLF10-deficient Vγ4^+^ γδ^27−^ cells might express higher amounts of surface CD5 than their WT counterparts. Indeed, we observed elevated CD5 expression on Vγ4^+^ γδ^27−^ cells of pLN from KO mice (Figure 5E; Figure S5 in Supplementary Material). It is noting that the considerable increase of CD5 was detected only in Vγ4^+^ γδ^27−^ cells, but not in other immune cells, including naive (CD44^lo^CD62L^hi^), effector (CD44^lo^CD62L^lo^), and memory (CD44^hi^CD62L^hi^ as central, CD44^hi^CD62L^lo^ as effector) phenotypes of CD4^+^ or CD8^+^ T cells (data not shown). Because CD5 is positively associated with the strength of TCR signaling that T cells received during their selection within the thymus (35), we next assessed the expression level of CD5 on thymic γδ T cells in neonates in which Vγ4^+^ γδ^27−^-17 cell maturation actively occurred (22). Interestingly, we observed considerably higher surface CD5 expression on mature (CD24^lo^CD44^hi^) thymic Vγ4^+^ γδ^27−^ cells under conditions of KLF10 deficiency, whereas the difference in CD5 was equivocal on the immature (CD24^hi^CD44^lo^) cells (Figures 5F,G). However, consistent with the peripheral observation (Figure S3E in Supplementary Material), TCR-triggered phosphorylation of ERK in neonatal thymic Vγ4^+^ γδ^27−^ cells was quite similar between both strains regardless of their maturation; ERK was hardly activated by TCR stimulation in Vγ4^+^ γδ^27−^ cells, contrary to the significant activation of ERK in γδ^27+^ cells (Figure 5H). These data collectively suggested that KLF10 deficiency resulted into CD5^int^Vγ4^+^CD127^hi^ γδ^27−^ cell development by controlling thymic maturation of Vγ4^+^ γδ^27−^ cells in neonates.

### Enhanced Thymic Maturation of Vγ4^+^ γδ^27−^-17 Cells in KLF10-Deficient Neonates

**Figure 6:**
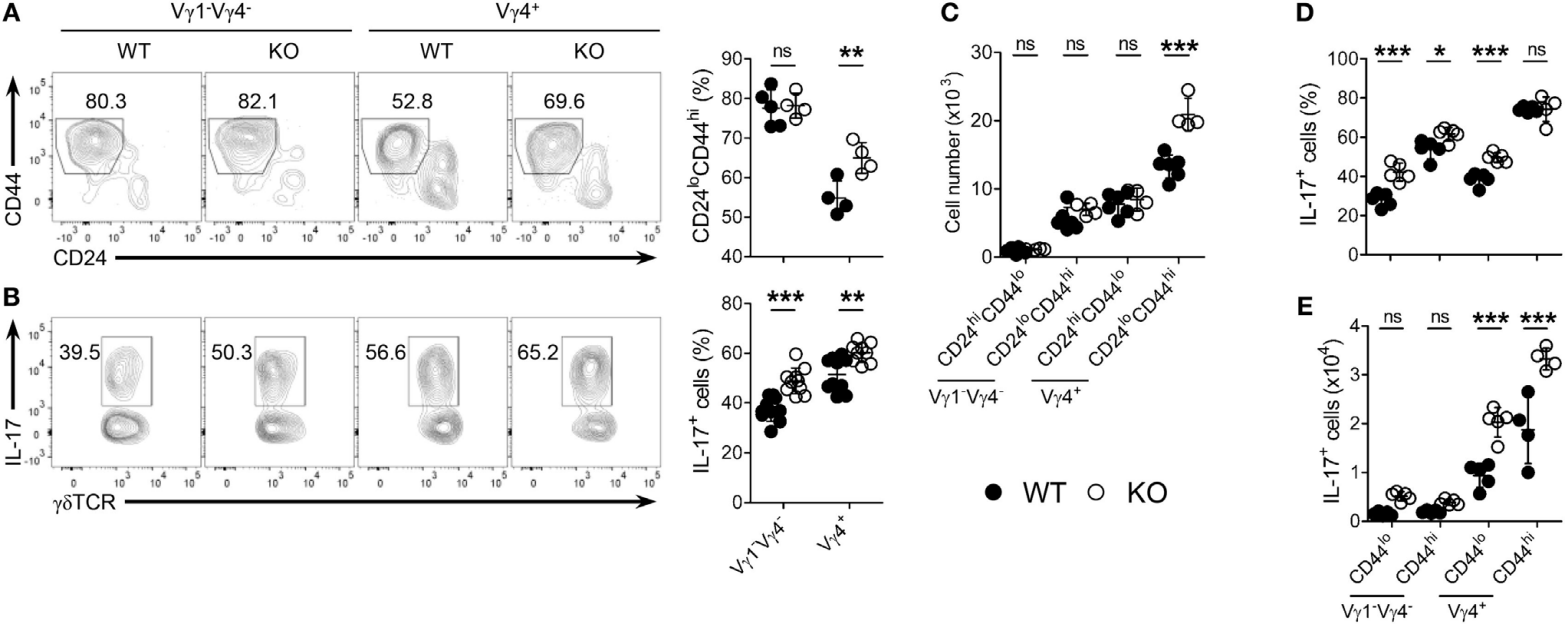
KLF10 deficiency leads to enhanced thymic maturation of Vγ4^+^ γδ^27−^-17 cells in neonates. **(A,B)** Density plot and percent of CD24^lo^CD44^hi^ cells **(A)** or IL-17^+^ cells [**(B)**; after stimulation with PMA plus ionomycin] among Vγ1^−^Vγ4^−^ or Vγ4^+^ γδ^27−^ cells in thymocytes from neonatal (day 4 after birth) wild-type (WT) and knockout (KO) mice (*n* ≥ 4 per group). **(C)** Absolute numbers of CD24^hi^CD44^lo^ and CD24^lo^CD44^hi^ cells in Vγ1^−^Vγ4^−^ or Vγ4^+^ γδ^27−^ cells obtained as in **(A)**. **(D,E)** Percent of IL-17^+^ cells among CD44^lo^ or CD44^hi^ cells of Vγ1^−^Vγ4^−^ or Vγ4^+^ γδ^27−^ cells **(D)** and their absolute numbers **(E)** obtained as in **(B)**. Numbers adjacent outlined areas of plots indicate percent of cells in each. Each symbol represents an individual mouse; error bars are the mean ± SD ns, non-significant; *P ≤ 0.05; **P ≤ 0.01; ***P ≤ 0.001. In **(C–E)**, two-way ANOVA followed by a Bonferroni posttest was used to determine significance. Data are representative of three independent experiments.

Next, we determined whether the selective expansion of CD5^int^Vγ4^+^ γδ^27−^-17 cells under KLF10 deficiency was developmental. As expected, Vγ4^+^ γδ^27−^ cell maturation was enhanced in the neonatal thymus of KO mice, as measured by the frequency of mature (CD24^lo^CD44^hi^) cells among Vγ4^+^ γδ^27−^ cells and their absolute number (Figures 6A,C). Intriguingly, frequencies of IL-17^+^ cells were higher in both Vγ1^−^Vγ4^−^ and Vγ4^+^ γδ^27−^ cells of KO neonatal thymus (Figure 6B) and this abundance was even observed at the immature (CD24^hi^CD44^lo^) stage (Figure 6D), indicating a general involvement of KLF10 in the IL-17-producing capacity of γδ^27−^ cells before thymic maturation. However, only Vγ4^+^ γδ^27−^-17 cells were significantly more abundant in the KO neonatal thymus at both immature and mature stages (Figure 6E), confirming the preferential restraint of KLF10 on Vγ4^+^ γδ^27−^-17 cell development.

### TCR-Dependent and -Independent Signaling Orchestrate KLF10 Execution Unique to Vγ4^+^ γδ^27−^ Thymic Development

**Figure 7:**
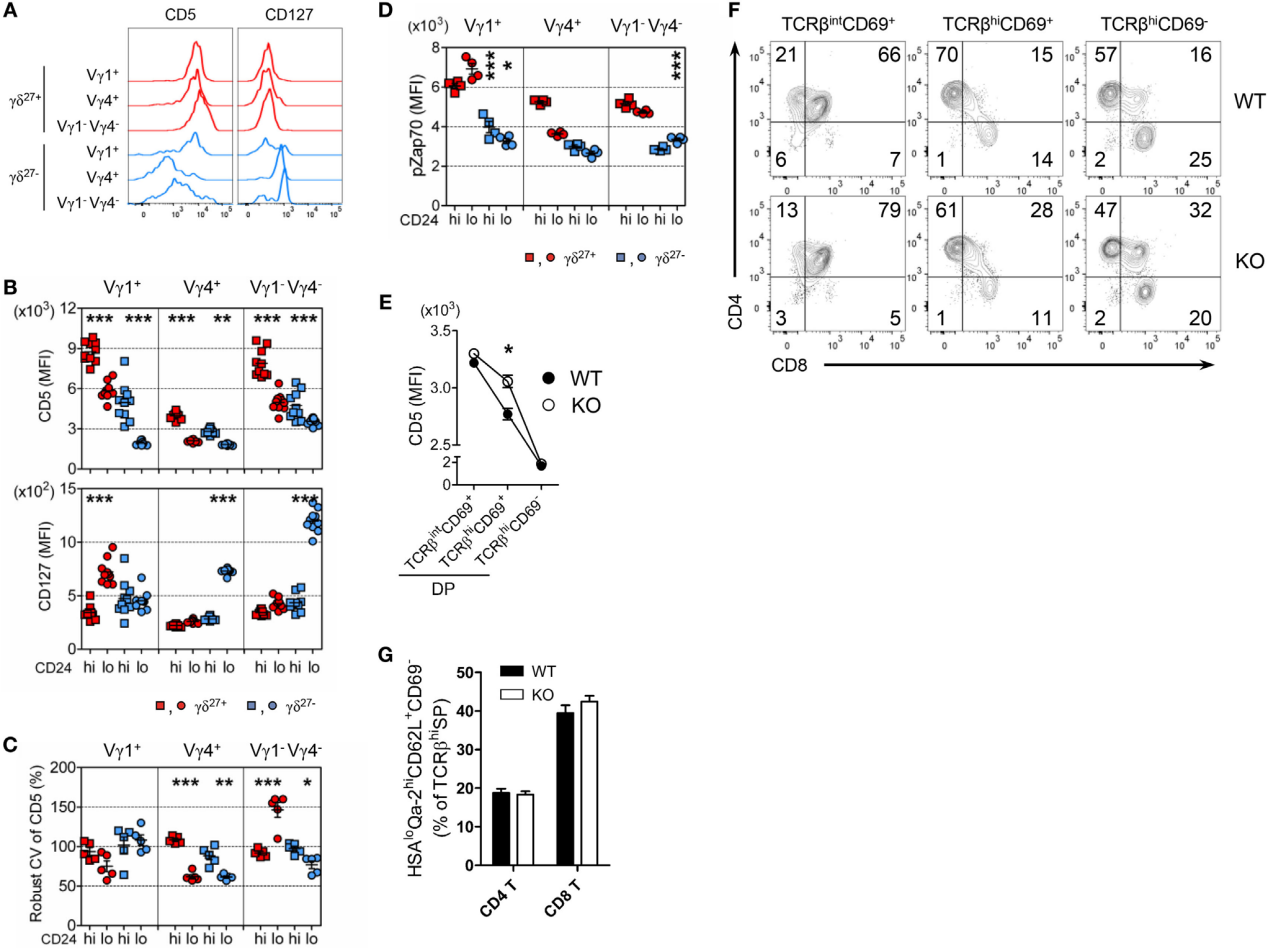
The uniqueness of Vγ4^+^ γδ^27−^ thymic development. **(A)** Half-offset histogram of CD5 and CD127 on Vγ1^+^, Vγ4^+^, and Vγ1^−^Vγ4^−^ subsets of γδ^27+^ or γδ^27−^ cells in peripheral lymph node cells from adult (6-week-old) WT mice. **(B–D)** MFI of CD5 and CD127 [**(B)**; *n* = 10], robust coefficient of variation (CV) of CD5 MFI [**(C)**; *n* = 5], and MFI of phosphorylated Zap70 [**(D)**; *n* = 4] on CD24^hi^ or CD24^lo^ cells of each subset [indicated as in **(A)**] from neonatal (day 3 after birth) thymocytes of WT mice. **(E–G)** MFI of CD5 on TCRβ^int^CD69^+^, TCRβ^hi^CD69^+^ and TCRβ^hi^CD69^−^ cells of CD4^+^CD8^+^ double-positive (DP) cells **(E)**, density plot of CD4 and CD8 expression on TCRβ^int^CD69^+^, TCRβ^hi^CD69^+^ and TCRβ^hi^CD69^−^ cells **(F)**, and percent of CD24^lo^Qa-2^hi^CD62L^+^CD69^−^ cells among CD4^+^ or CD8^+^ TCRβ^hi^ cells **(G)** in thymocytes from WT and KO mice (*n* ≥ 4). Data **(E,G)** are mean ± SD. Each symbol **(B–D)** represents an individual mouse; error bars are the mean ± SD. *P ≤ 0.05; **P ≤ 0.01; ***P ≤ 0.001. Data are representative of at least three independent experiments.

Consistent with the difference in CD5 expression on γδ subsets in the periphery (Figures 5C and 7A; Figure S5 in Supplementary Material), CD5 expression on each γδ thymic subset was unique and distinct based on the expression of Vγ chains and CD27 (Figure 7B). These results might suggest that different strength of TCR signal is required for thymic development of γδ subsets (11, 26). Regardless of CD27, the intensity of CD5 expression on immature Vγ4^+^ subsets was at a low level and distinct from that of immature Vγ1^+^ or Vγ1^−^Vγ4^−^ subsets, suggesting a requirement for weaker TCR signaling for thymic emergence of Vγ4^+^ cells (Figure 7B). We noted that immature Vγ4^+^ γδ^27−^ thymocytes expressed the lowest level of CD5, which was similarly maintained in the periphery (Figure 7A).

We observed that thymic maturation induced a general decrease in CD5 expression on γδ T cells (Figure 7B). However, the CV of CD5 dramatically decreased in only Vγ4^+^ thymocytes after maturation (Figure 7C), suggesting their restricted spectrum of TCR-signal mode. To determine the basal activity of TCR signaling, we directly assessed intracellular levels of pZap70 in each γδ subset (Figure 7D). Interestingly, in contrast to the Vγ chain-specific clustering of CD5 MFI (Figure 7B), the level of pZap70 in immature γδ subsets clustered according to the expression of CD27 (Figure 7D), suggesting that each immature Vγ subset possessed basal TCR signaling that was influenced by CD27 costimulation (14, 32). Nonetheless, the level of pZap70 in immature Vγ4^+^ γδ^27−^ thymocytes was low, similar to that in immature Vγ1^−^Vγ4^−^ γδ^27−^ thymocytes, and then slightly decreased in the Vγ4^+^ γδ^27−^ subset and increased in the Vγ1^−^Vγ4^−^ γδ^27−^ subset after maturation; thus, a Vγ4^+^ γδ^27−^ subset could acquire a relatively lower activity of basal TCR signaling (Figure 7D). Together, these data emphasized a TCR-signaling modality unique to the emergence and maturational transition of a Vγ4^+^ γδ^27−^ subset, endorsing a weak TCR-signaling requirement for innate-like γδ-17 differentiation (10, 36). It is important to note that *Klf10* could be induced by both TCR-dependent and -independent signaling pathways (Figures 3F,H), which were differently engaged in γδ development (24). On the other hand, CD127 (IL-7Rα) was abruptly induced in Vγ4^+^ or Vγ1^−^Vγ4^−^ γδ^27−^ subsets after thymic maturation (Figure 7B), consistent with the notion of mature stage-specific acquisition of cytokine receptor-mediated regulation of γδ effector differentiation (25). Therefore, a series of weak TCR-signaling engagements with subsequent initiation of cytokine signaling seemingly cooperate for the function of KLF10 specific to Vγ4^+^ γδ^27−^ thymic development by primarily fine-tuning KLF10 transcription.

Transcriptional profiling of thymic Vγ subsets revealed that immature Vγ4^+^ subsets are distinct from other immature Vγ subsets (Vγ1^+^, Vγ1.1^+^Vδ6.3^+^, and Vγ5^+^) but, interestingly, closely similar to CD4^+^CD8^+^ double-positive (DP) CD69^+^ cells of the αβ lineage, based on low expression of genes involved in metabolism and energy production (25). Of note, a gene constellation browser publicly provided by the Immunological Genome Project (ImmGen; www.immgen.org) reported that *Klf10* in γδ T cells was closely correlated with genes encoding metabolic molecules, which showed lower expression in the immature Vγ4^+^ subset than in other Vγ subsets (data not shown) (25, 37). When we assessed the expression of CD5 on DP cells, KO DP cells transiently displayed relatively higher CD5 expression than their WT counterparts at post-positive selection, but not at the fully matured (TCRβ^hi^CD69^–^) stage (Figure 7E), accompanied by a delayed CD4 lineage choice (Figure 7F). However, maturation of CD4 and CD8 T cells was normal in both strains, as measured by the percentage of CD24^lo^Qa-2^hi^CD62L^+^CD69^−^ cells among TCRβ^hi^ CD4^+^ or CD8^+^ cells (Figure 7G). Thus, these data suggested that the putative mechanism involved in mature stage-specific alteration of surface CD5 on the Vγ4^+^ γδ^27−^ thymic subset by KLF10 deficiency (Figures 5F,G) might be related to a metabolic process that is common to both Vγ4^+^ subsets and DP CD69^+^ cells.

### KLF10 Intrinsically Regulates the Development of Vγ4^+^ γδ^27−^-17 Cells

**Figure 8:**
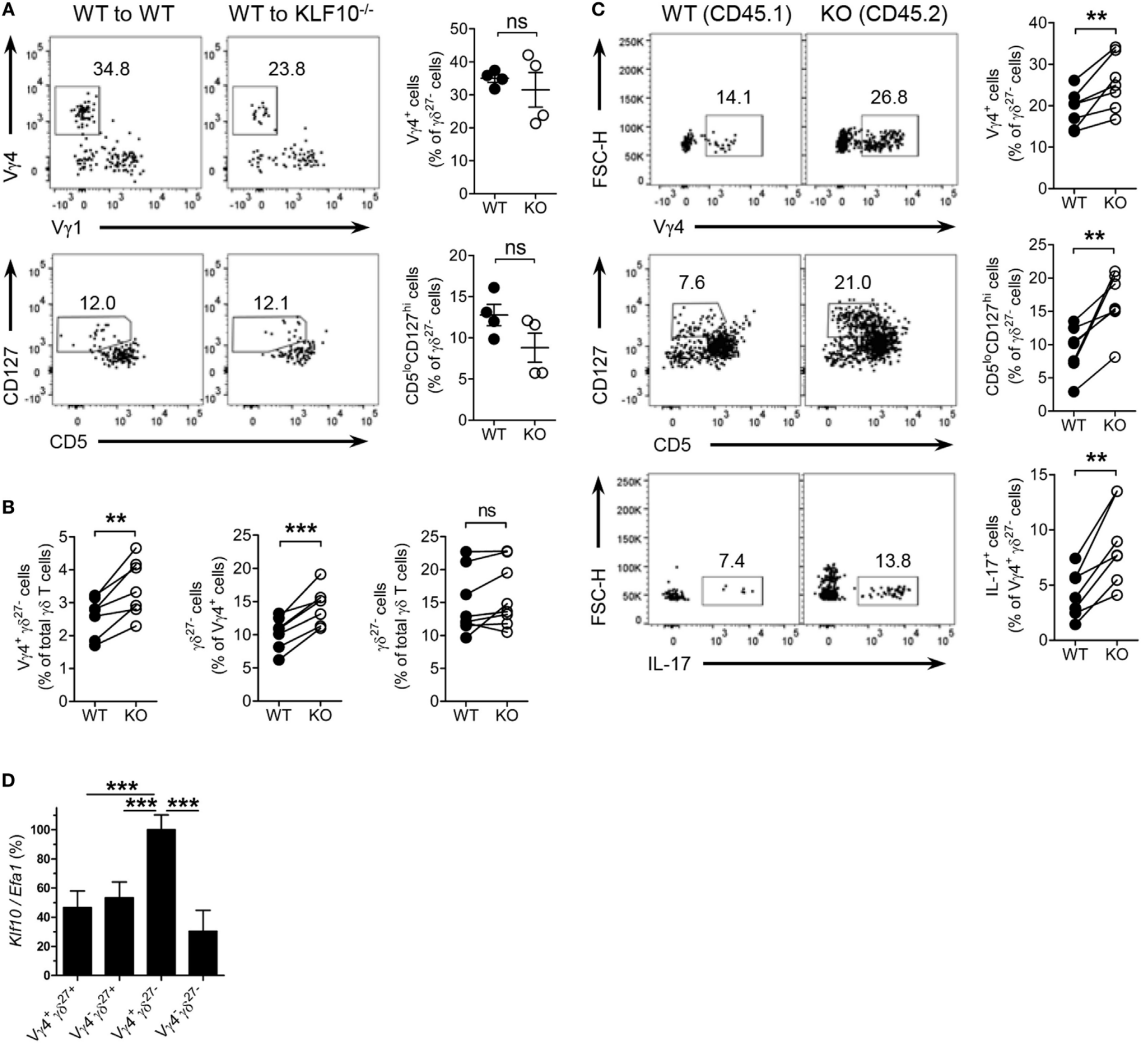
KLF10 restraint of innate-like Vγ4^+^ γδ^27−^-17 cells is cell-intrinsic. **(A)** Dot plot and percent of Vγ4^+^Vγ1^−^ cells (top) and CD5^lo^CD127^hi^ cells (bottom) among peripheral lymph node (pLN) γδ^27−^ cells of CD45.2 WT or KO mice (*n* = 4), irradiated and reconstituted with CD45.1 WT bone marrow (BM) cells and then assessed by flow cytometry at least 12 weeks later, gated on CD45.1^+^ γδ^27−^ cells. **(B,C)** CD45.1/2 WT mice (*n* ≥ 3) were irradiated and reconstituted with a mixture of CD45.1 WT BM plus CD45.2 KO BM cells at ratio 1:1 and then pLN cells were analyzed by flow cytometry at least 12 weeks later. **(B)** Percent of Vγ4^+^ γδ^27−^ cells (left) or γδ^27−^ cells (right) among WT and KO total γδ T cells or, γδ^27−^ cells (middle) among WT and KO Vγ4^+^ cells. **(C)** Dot plot (left) and percent (right) of Vγ4^+^ (top) or CD5^lo^CD127^hi^ cells (middle) among WT and KO γδ^27−^ cells, or IL-17^+^ cells (bottom) among WT and KO Vγ4^+^ γδ^27−^ cells. **(D)** Real-time reverse transcription PCR analysis of *Klf10* expression in sorted Vγ4^+^ γδ^27+^, Vγ4^−^ γδ^27+^, Vγ4^+^ γδ^27−^, and Vγ4^−^ γδ^27−^ cells from pooled pLN and spleen of WT mice (*n* = 10 per group), normalized to the housekeeping gene *Efa1* and presented as the percent of maximum expression of *Klf10*. Data **(A,D)** are mean ± SD. Each symbol represents an individual recipient mouse. A line connects WT-derived cells to KO-derived cells that developed within the same recipient mouse. ns, non-significant; **P ≤ 0.01; ***P ≤ 0.001. Data are representative of two independent experiments **(A,D)** or are pooled from two independent experiments **(B,C)**.

Finally, we examined whether KLF10 extrinsically or intrinsically controlled homeostasis of Vγ4^+^ γδ^27−^-17 cells. We reconstituted irradiated CD45.2^+^ WT or KO mice with congenic WT BM cells and analyzed CD45.1^+^ γδ^27−^ cells after at least 12 wks (Figure S6B in Supplementary Material). Reconstitution of Vγ4^+^ γδ^27−^ cells and CD5^lo^CD127^hi^ γδ^27−^ cells in KO recipients was achieved at a level comparable to that in WT recipients, excluding the hematopoietic system-extrinsic effect of KLF10 on the homeostasis of Vγ4^+^ γδ^27−^ 17 cells (Figure 8A). By contrast, mixed BM chimera experiments in which a 1:1 mixture of CD45.1^+^ WT and CD45.2^+^ KO BM cells was injected into lethally irradiated CD45.1/2^+^ WT mice (Figure S8C in Supplementary Material) showed a higher proportion of KO BM-derived cells among total CD3ε^+^ cells (data not shown). This suggested that KLF10-deficient BM cells outcompeted their WT counterparts during reconstitution of hematopoietic-derived cells, with the fact that KLF10 transcripts are at high level in long- and short-term repopulating hematopoietic stem cells (www.immgen.org).

We next investigate whether γδ cell-intrinsic KLF10 role is responsible for the restraint of Vγ4^+^ γδ^27−^ cells. In the mixed BM chimera setting, a proportion of Vγ4^+^ γδ^27−^ cells in KO BM-derived γδ T cells was relatively higher than that of their WT counterpart (Figure 8B). Although the total γδ T cells from both WT and KO BM cells contained γδ^27−^ cells similarly, the KO BM-derived Vγ4^+^ cells generated γδ^27−^ cells more than their WT counterpart did (Figure 8B); as expected, there were comparable proportions of γδ^27−^ cells in Vγ4^−^ cells and of Vγ4^+^ cells in γδ^27+^ cells between the both origins (Figure S8D in Supplementary Material). Indeed, KO BM-derived γδ^27−^ cells contained higher proportions of Vγ4^+^ cells and CD5^lo^CD127^hi^ cells than their WT BM-derived counterparts (Figure 8C). Of note, KO BM-derived Vγ4^+^ γδ^27−^ cells had greater frequencies of IL-17^+^ cells (Figure 8C). We also found a higher level of *Klf10* expression in Vγ4^+^ γδ^27−^ cells compared to the other γδ subsets (Figure 8D), possibly supporting the preferential engagement of KLF10 for the homeostasis of Vγ4^+^ γδ^27−^ cells. Collectively, these results suggest that KLF10 serves as an intrinsic negative regulator to constrain Vγ4^+^ γδ^27−^-17 cells and their production of IL-17.

## Discussion

Early studies revealed that KLF10-deficient mice had defective Treg cell generation under inflammatory conditions, emphasizing the role of KLF10 as a TF in the balance between Treg and Th17 cell differentiation (1–3). Although KLF10, previously named TIEG-1 (TGF-β-induced early gene-1), can be rapidly induced in CD4 T cells after TGF-β stimulation and then functions to maintain the activation of TGF-β/Smad signaling pathway (2), we found that KLF10 transcription did not respond to TGF-β in γδ^27−^ cells (data not shown), indicating that KLF10 function in γδ-17 cells was irrelevant to TGF-β/Smad signaling for γδ-17 cell development (5). Intriguingly, by analyzing these KO mice under steady-state conditions (unimmunized and specific pathogen-free), we have identified KLF10 as a critical negative regulator of development and homeostasis of Vγ4^+^ γδ^27−^ cells. KLF10-deficient mice exhibited a spontaneous and selective augmentation of Vγ4^+^ γδ-17 cells with normal frequencies of IL-17-producing TCRβ^+^ cells such as Th17, Tc-17 (IL-17^+^CD8^+^TCRβ^+^) and *i*NKT-17 (IL-17^+^CD1d-tet^+^TCRβ^+^) cells, as well as Treg cells, suggesting a novel function of KLF10 unique to innate-like γδ-17 cells. Meanwhile, the slight increase of IFN-γ^+^ γδ^27+^ cells in pLN of KO mice might demand further investigation.

Robust expansion of innate-like γδ-17 cells under lymphopenic conditions is completely dependent on homeostatic cytokine IL-7 but not MHC recognition (15, 16). However, because of cellular competition for trophic cytokines or space (15), γδ T cell homeostatic expansion was inhibited by αβ T cells after transfer of pLN cells into lymphopenic mice. Interestingly, we found that KLF10 deficiency allowed γδ^27−^ cells to overcome the competitive inhibition by αβ T cells, reflecting the increased sensitivity of IL-7 signaling in KO γδ^27−^ cells. This was confirmed by the hyper-responsiveness of KO γδ^27−^ cells to exogenous IL-7 stimulation with increased STAT3 activation. KO γδ^27−^ cells also hyper-responded to IL-1β plus IL-23 stimuli. Of note, KLF10 transcription in γδ^27−^ cells was dramatically induced by IL-6 (data not shown), IL-7, or IL-1β plus IL-23, which are known to trigger STAT3 activation to induce RoRγt expression (9, 38, 39), suggesting that KLF10 was a negative regulator of a STAT3-RoRγt axis in γδ-17 cells. Clearly, further studies are needed to determine the target genes, interacting signal proteins, and post-translational modifications of KLF10 to discern how KLF10 contributes to cytokine-signaling pathways in γδ-17 cells. On the other hand, KLF10 was closely correlated with cell division control protein 42 homolog and Fas apoptotic inhibitory molecule in γδ T cells according to a gene constellation view by ImmGen (data not shown) (37), suggesting a direct link between KLF10 and prosurvival proteins such as Bcl-2 and Bcl-xL, which are upregulated by IL-7 in γδ-17 cells (16). Therefore, it is necessary to explore whether KLF10 directly engages in entry into the cell cycle and the intrinsic cell death pathway of γδ-17 cells (16, 40).

The increased IL-7 signaling sensitivity of KO γδ^27−^ cells was independent of Vγ4 under conditions of strong reliance on IL-7 (lymphopenic condition or direct treatment with IL-7), which is different from the Vγ4^+^ subset-specific γδ^27−^ cell enrichment observed in KO mice. IL-7Rα expression was not only similar between Vγ4^+^ and Vγ1^−^Vγ4^−^ γδ^27−^ subsets, both of which are main producers of innate IL-17 among γδ T cells, but was also unchanged by KLF10 deficiency (data not shown). These data suggest that Vγ4^−^ γδ^27−^ cells, presumably Vγ1^−^Vγ4^−^ γδ^27−^ subsets, are under homeostatic control by certain factors counteracting the effect of an active IL-7R-KLF10 signal axis (15). These factors could be directly or indirectly involved in the downstream of IL-7R signaling. Alternatively, considering that the steady-state level of KLF10 transcripts was preferentially higher in Vγ4^+^ γδ^27−^ cells than in other γδ subsets, we could postulate that Vγ1^−^Vγ4^−^ γδ^27−^ subset-specific factors might dampen the inhibitory effect of KLF10 on IL-7-mediated homeostasis by downregulating KLF10 transcription.

Our analysis of surface CD5 expressed on emergent immature γδ thymocytes clearly indicated discrete TCR-signal engagements for Vγ chains. Immature Vγ4^+^ thymocytes adopted relatively low and narrow-ranged CD5 expression regardless of CD27 expression, seemingly consistent with the ligand-independent signaling of Vγ4^+^Vδ5^+^ TCR (10). This also suggests that Vγ4^+^ subsets may require relatively weak TCR-signal engagement for thymic emergence compared with other Vγ subsets, in support of the previous report that Vγ6^+^ thymocytes may depend on strong TCR signaling (11, 26, 41). Indeed, the surface CD5 expression of the peripheral Vγ4^+^ γδ^27−^ subset was apparently lower than that of the Vγ1^−^Vγ4^−^ (and presumably Vγ6^+^) γδ^27−^ subset (17). Most of all, we confirmed that innate-like γδ-17 cells received relatively weak TCR-signal strength during thymic development by identifying IL-17-committed γδ T cells as CD5^lo^CD127^hi^ γδ^27−^ cells predominantly composed of Vγ4^+^ and Vγ1^−^Vγ4^−^ subsets.

Seemingly, the lower expression of CD5 on immature Vγ4^+^ thymocytes and the subsequent super-induction of IL-7Rα on γδ^27−^ thymocytes upon maturation might indicate the initial engagement of weak TCR-signal strength for the Vγ4 wave, followed by heavy reliance upon IL-7 signaling for functional maturation during development (9, 25, 26). Considering that KLF10 transcription could be induced by either TCR or IL-7 signaling, we could anticipate that expression of the KLF10 transcript would be relatively low in immature Vγ4^+^ thymocytes compared with other thymic immature Vγ subsets and then increase after maturation; indeed, the expected results were obtained from the public data resources of ImmGen (37). Interestingly, in contrast to KLF10, Sox13 transcription is suppressed by both TCR (36) and IL-7 stimuli (data not shown). Above all, Sox13 is highly expressed in thymic γδ progenitors as a γδ-lineage specific marker, but after maturation dramatically decreased with relatively higher expression in γδ-17 thymocytes than in γδ-IFN-γ thymocytes (36, 42). Of note, there is evidence that Sox13 is essential for the development of Vγ4^+^ γδ-17 cells, as it is abundant in Vγ4^+^ rather than in Vγ4^−^ thymocytes at an immature stage (22, 37, 43). However, we found similar levels of Sox13 transcripts in the peripheral Vγ4^+^ γδ^27−^ subset between WT and KO mice (data not shown), suggesting that Sox13 may not be associated with the selective effect of KLF10 deficiency on Vγ4^+^ γδ^27−^ cell development (22, 43). This notwithstanding, the lower levels of KLF10 and higher levels of Sox13 in immature Vγ4^+^ thymocytes, with lower CD5 expression, imply engagement of weak-TCR-signal strength in Vγ4^+^ γδ-17 thymic emergence. Furthermore, the more substantial involvement of KLF10 and Sox13 in Vγ4^+^ γδ-17 cell development reinforced distinct developmental requirements for Vγ4^+^ and Vγ6^+^ γδ-17 cells (11, 26, 43).

We showed that the intensity of surface CD5 was mildly but significantly increased in both thymic mature Vγ4^+^ γδ^27−^ cells and TCRβ^hi^CD69^+^ DP cells of KO mice. This analogous CD5 alteration by KLF10 deficiency could support the idea of close similarity between Vγ4^+^ cells and DP cells at an immature stage (25). The close relationship between KLF10 and genes involved in metabolic processes, whose expression was lower in these two populations, provided mechanistic insight into thymic maturation of CD5^int^Vγ4^+^ γδ-17 cells under KLF10 deficiency. Moreover, a recent report revealed that KLF10 binds to a nutritional regulatory element in the promoter region of SREBP-1c that is critical for glucose and lipid metabolism (44). On the other hand, maturational transition of γδ T cells seems quite similar to “negative selection” in the light of the facts that surface CD5 is commonly reduced after maturation of γδ T cells and the CD5 level correlates with the strength of TCR signal initially perceived (35, 45, 46).

We suggest that thymic programming for the generation of Vγ4^+^ γδ-17 cells is negatively regulated by KLF10, with many questions remaining to be answered. In particular, clear elucidation of the quantitatively and qualitatively distinct engagements of the TCR signal, whose role in γδ-17 effector decisions is still controversial, will advance identification of the developmental mechanism of Vγ4^+^ γδ-17 cells under the transcriptional control of KLF10 (26); it is possible that Vγ chains transmit distinct TCR signals caused by differences in either intrinsic modality or extrinsic factors, such as selecting ligands or that a unique TCR signal (strength and duration) may trigger the expression of genes encoding certain Vγ chains (10). Moreover, determining whether KLF10 is associated with signal circuits of inherited (or intrinsic) TFs specific to Vγ4^+^ γδ-17 cell development and with the timing of generation of the Vγ4 wave will give insight into γδ effector subset diversification (24, 47, 48).

## Ethics Statement

All animals were bred and maintained under specific pathogen-free conditions at the Institute of Laboratory Animal Resource Seoul National University and treated in accordance with institutional guidelines that were approved by the Institutional Animal Care and Use Committee (SNU-140930-4-1).

## Author Contributions

C-HY conceived the idea. C-HY and GK designed the experiments and wrote the manuscript. GK, MG, SK, KK, Y-CK, and CK performed the experiments. GK interpreted the data. J-HC, W-KL, K-DS, HC, Y-MP, and SH provided critical comments. All authors contributed to discussion of the results followed by writing and reviewing the manuscript.

## Conflict of Interest Statement

The authors declare that the research was conducted in the absence of any commercial or financial relationships that could be construed as a potential conflict of interest.
