# Supplementary material for: Transcription Factor KLF10 Constrains IL-17-Committed Vγ4+ γδ T Cells
Source: Front Immunol. 2018 Feb 28;9:196. doi: 10.3389/fimmu.2018.00196 (PMC5835516; doi:10.3389/fimmu.2018.00196)
Supplement: Supplementary file 1 [file Data_Sheet_1.PDF]

Supplementary Figure 1

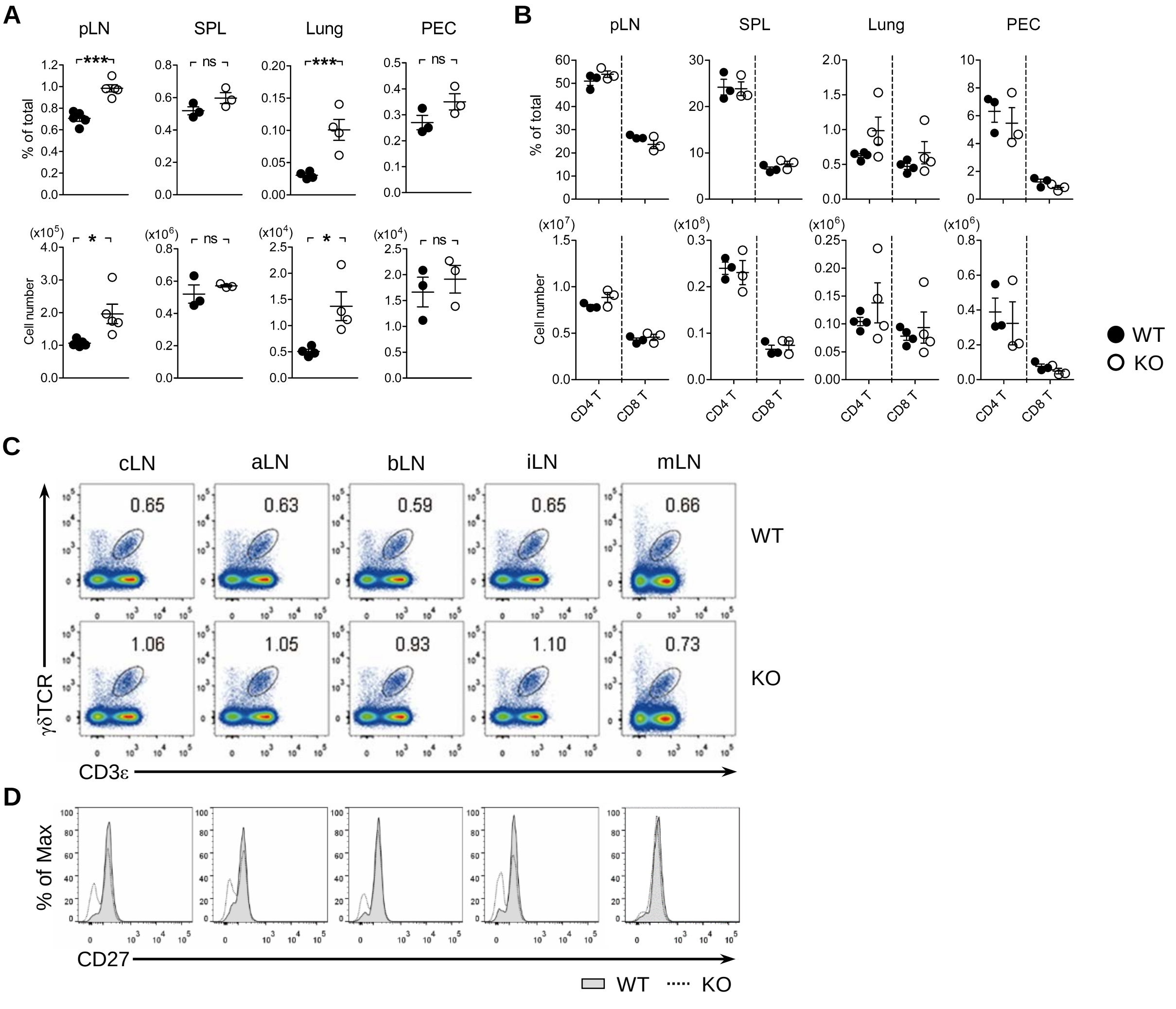

Supplementary Figure 1. γδ T cells, but not αβ T cells are augmented in KLF10-deficient mice. (**A**,**B**) Frequency and absolute number of γδ T cells (**A**) and αβ T cells (**B**) in pLN, spleen (SPL), lung and peritoneal exudate cells (PEC) obtained from wild-type (WT) and KLF10-deficient (KO) mice (n ≥ 3 per group). Each symbol represents an individual mouse; error bars are the mean ± s.d. ns, non-significant; * $P \leq 0.05$; *** $P \leq 0.001$. (**C**,**D**) Pseudocolor plot of frequency of γδ T cells (**C**) and overlayed histogram of CD27 expression on γδ T cells (**D**) in cervical (cLN), axillary (aLN), brachial (bLN), inguinal (iLN) and mesenteric (mLN) lymph nodes from both strains (n = 4 per group). Numbers adjacent outlined areas of the plot indicate percent of cells in each. Data are representative of at least three independent experiments.
