# Supplementary material for: Transcription Factor KLF10 Constrains IL-17-Committed Vγ4+ γδ T Cells
Source: Front Immunol. 2018 Feb 28;9:196. doi: 10.3389/fimmu.2018.00196 (PMC5835516; doi:10.3389/fimmu.2018.00196)
Supplement: Supplementary file 2 [file Data_Sheet_2.PDF]

Supplementary Figure 2

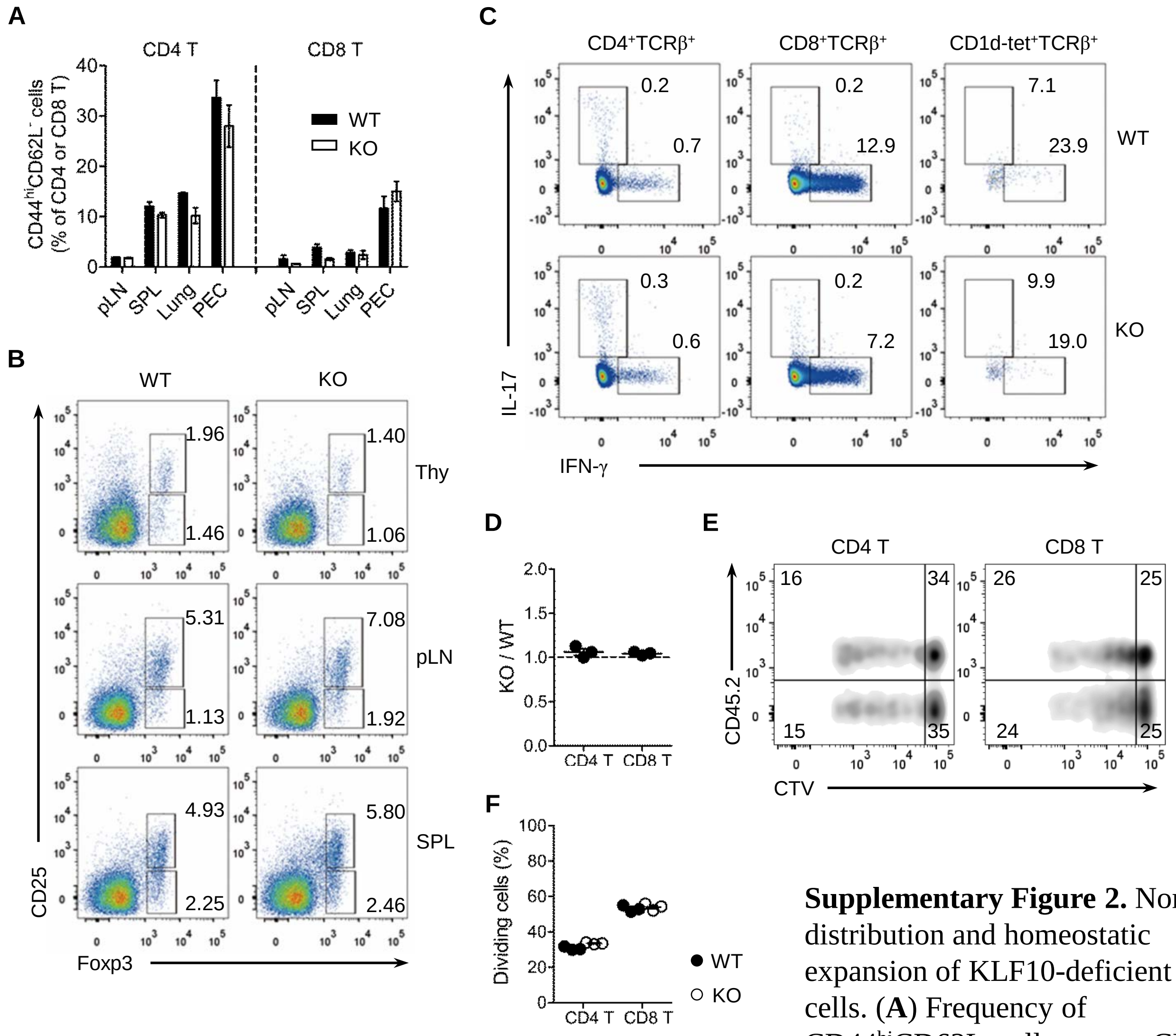

Supplementary Figure 2. Normal distribution and homeostatic expansion of KLF10-deficient αβ T cells. (**A**) Frequency of $CD44^{hi}CD62L^{-}$ cells among $CD4^{+}$ or $CD8^{+}$ T cells of pLN, spleen (SPL), lung and peritoneal exudate cells (PEC) obtained from wild-type (WT) and KLF10-deficient (KO) mice (n = 5 per group). Data are the mean ± s.d. (**B**) Pseudocolor plot of $Foxp3^{+}$ Tregs among adult thymus (Thy), pLN and SPL obtained from both strains (n = 3 per group); plots are gated on $CD4^{+}TCR\beta^{+}$ cells. (**C**) Pseudocolor plot of IL-17- or IFN-γ-competent cells among $CD4^{+}$ T ($CD4^{+}TCR\beta^{+}$), $CD8^{+}$ T ($CD8^{+}TCR\beta^{+}$) or invariant NKT (CD1d-tet$^{+}TCR\beta^{+}$) cells in pLN cells from both strains after stimulation with PMA and ionomycin (n =3 per group). (**D**-**F**) The ratio of KO to WT CD4 T or CD8 T cells (**D**), homeostatic expansion of WT versus KO CD4 T or CD8 T cells (**E**), and percentage of dividing cells among CD4 T or CD8 T cells of both strains (**F**) from pLN cells of Rag-1-deficient mice, co-injected with CTV-labeled CD45.1 WT and CD45.2 KO pLN cells at ratio of 1:1 and then analyzed by flow cytometry after 5 d. Each symbol represents an individual mouse; error bars are the mean ± s.d. Numbers adjacent outlined areas or quadrants of the plot indicate percent of cells in each. Data are representative of at least two independent experiments.
