# Supplementary material for: Transcription Factor KLF10 Constrains IL-17-Committed Vγ4+ γδ T Cells
Source: Front Immunol. 2018 Feb 28;9:196. doi: 10.3389/fimmu.2018.00196 (PMC5835516; doi:10.3389/fimmu.2018.00196)
Supplement: Supplementary file 3 [file Data_Sheet_3.PDF]

Supplementary Figure 3

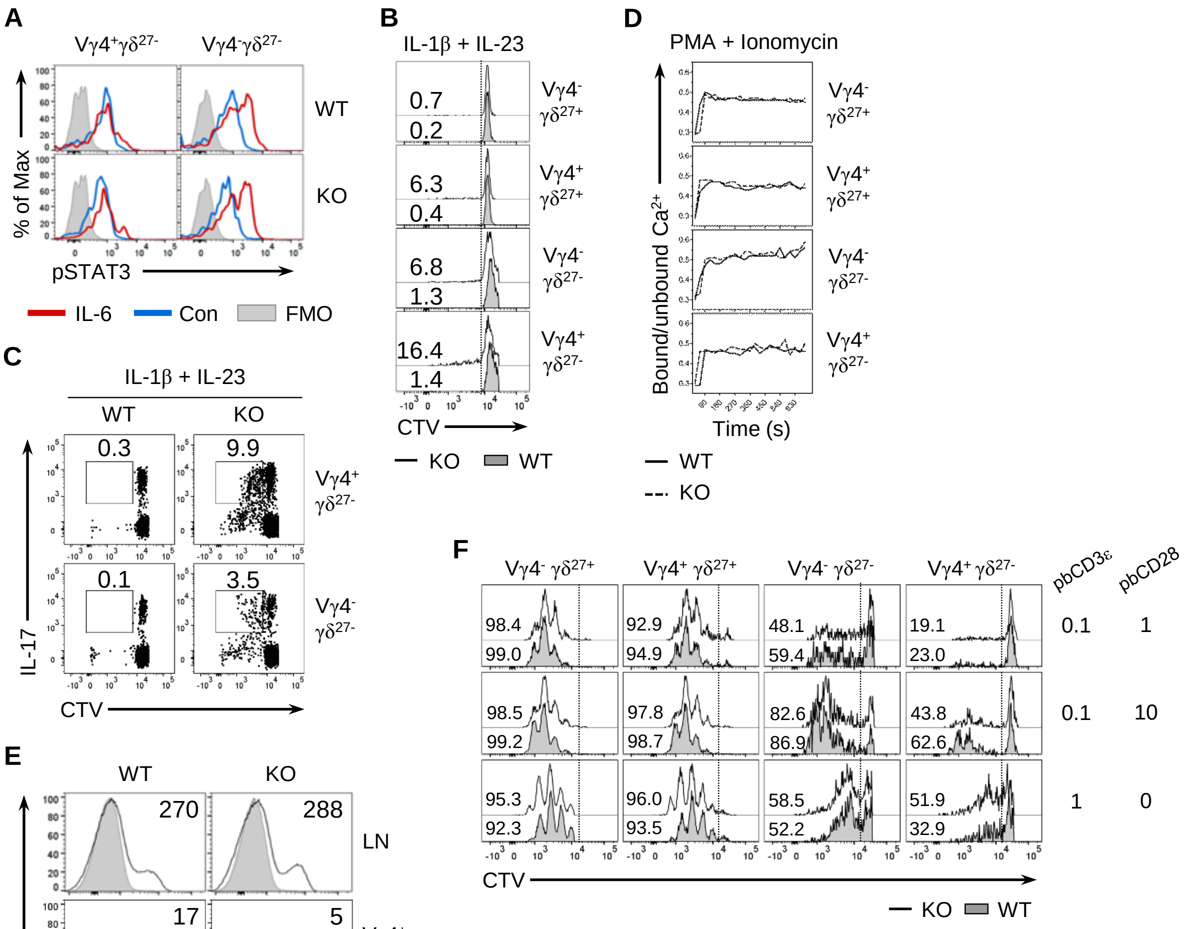

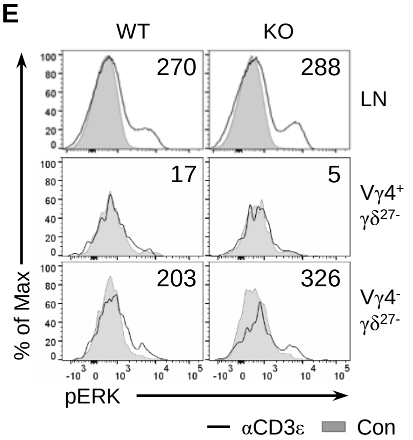

Supplementary Figure 3. KLF10 is involved in γδ T cell response to cytokine stimuli. **(A)** Overlayed histogram of phosphorylated STAT3 in Vγ4^{+} γδ^{27-} or Vγ4^{-} γδ^{27-} cells from pooled WT or KO pLN cells (n ≥ 3 per group) cultured with IL-6 (20 ng/ml) for 30 min and assessed by flow cytometry. Con, control; FMO, fluorescence minus one. (**B**,**C**) Half-offset histogram of proliferation (**B**) and dot plot of IL-17 expression (**C**; after stimulation with PMA plus ionomycin) of γδ T cells (sorted as in **Fig. 3B**), CTV-labeled and cultured with IL-1β (10 ng/ml) plus IL-23 (20 ng/ml) for 3 d. Numbers adjacent outlined areas (**C**) indicate percent of cells in each. (**D**) Intracellular Ca^{2+} mobilization (as in **Fig. 3I**) in γδ T cells, stimulated with PMA (100 ng/ml) plus ionomycin (500 ng/ml) and then assayed over 10 min (n ≥ 4 per group). (**E**) Overlayed histogram of phosphorylated ERK in Vγ4^{+} or Vγ4^{-} γδ $^{27-}$ cells from pLN cells of WT and KO mice (n ≥ 3 per group), stimulated with soluble anti-CD3ε (10 μg/ml). Numbers indicate MFI variation between cells cultured with or without anti-CD3ε. (**F**) Half-offset histogram of proliferation of γδ T cells (sorted as in **Fig. 3B**), CTV-labeled and cultured on plated-bound anti-CD3ε (0.1, 1 μg/ml) and anti-CD28 (0, 1, 10 μg/ml) for 3 d. Numbers in **B**,**F** indicate percent of dividing cells. Data are representative of at least three independent experiments (**B**,**C**,**E**) or two independent experiments (**A**,**D**,**F**).
