# Supplementary material for: Transcription Factor KLF10 Constrains IL-17-Committed Vγ4+ γδ T Cells
Source: Front Immunol. 2018 Feb 28;9:196. doi: 10.3389/fimmu.2018.00196 (PMC5835516; doi:10.3389/fimmu.2018.00196)
Supplement: Supplementary file 4 [file Data_Sheet_4.PDF]

Supplementary Figure 4

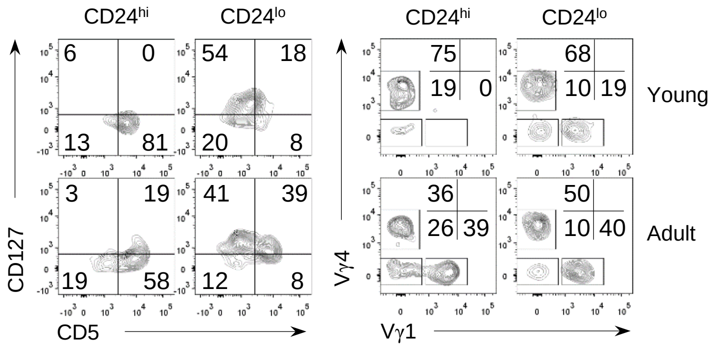

Supplementary Figure 4. The frequency of CD5^{hi}Vγ1^{+} cells among thymic γδ^{27-} cells were higher in adult mice compared to the young. Thymocytes of 2- (Young) and 8-week-old (Adult) wild-type mice (n ≥ 3 per each) were gated on CD3ε^{+}γδTCR^{+}CD27^{-} cells. Data are representative of two independent experiments.
