# Supplementary material for: Transcription Factor KLF10 Constrains IL-17-Committed Vγ4+ γδ T Cells
Source: Front Immunol. 2018 Feb 28;9:196. doi: 10.3389/fimmu.2018.00196 (PMC5835516; doi:10.3389/fimmu.2018.00196)
Supplement: Supplementary file 5 [file Data_Sheet_5.PDF]

Supplementary Figure 5

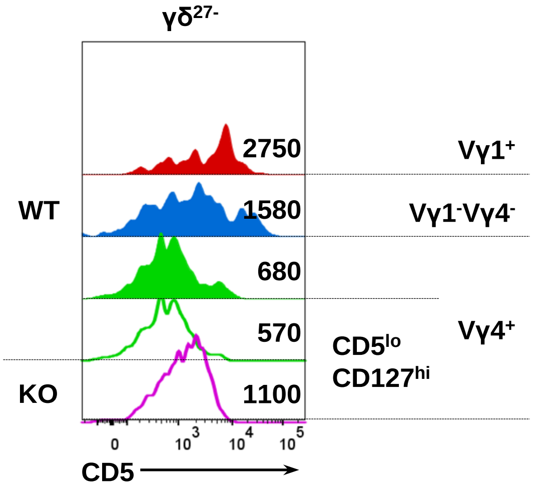

Supplementary Figure 5. The comparison of CD5 expression on Vγ1$^+$ (red filled), Vγ1$^-$Vγ4$^-$ (blue filled), Vγ4$^+$ (green filled), CD5^{lo}CD127^{hi}Vγ4$^+$ cells (green line) of WT $\gamma\delta^{27-}$ cells and CD5^{lo}CD127^{hi}Vγ4$^+$ cells (violet line) of KO $\gamma\delta^{27-}$ cells. pLN cells from WT and KO mice were analyzed by flow cytometry, gated on CD3$\varepsilon^+\gamma\delta$TCR$^+$CD27$^-$ cells. CD5^{lo}CD127^{hi} cells were defined as in **Fig. 4B**. Numbers indicate geometric MFI of CD5. Data are representative of three independent experiments.
