# Supplementary material for: Transcription Factor KLF10 Constrains IL-17-Committed Vγ4+ γδ T Cells
Source: Front Immunol. 2018 Feb 28;9:196. doi: 10.3389/fimmu.2018.00196 (PMC5835516; doi:10.3389/fimmu.2018.00196)
Supplement: Supplementary file 6 [file Data_Sheet_6.PDF]

Supplementary Figure 6

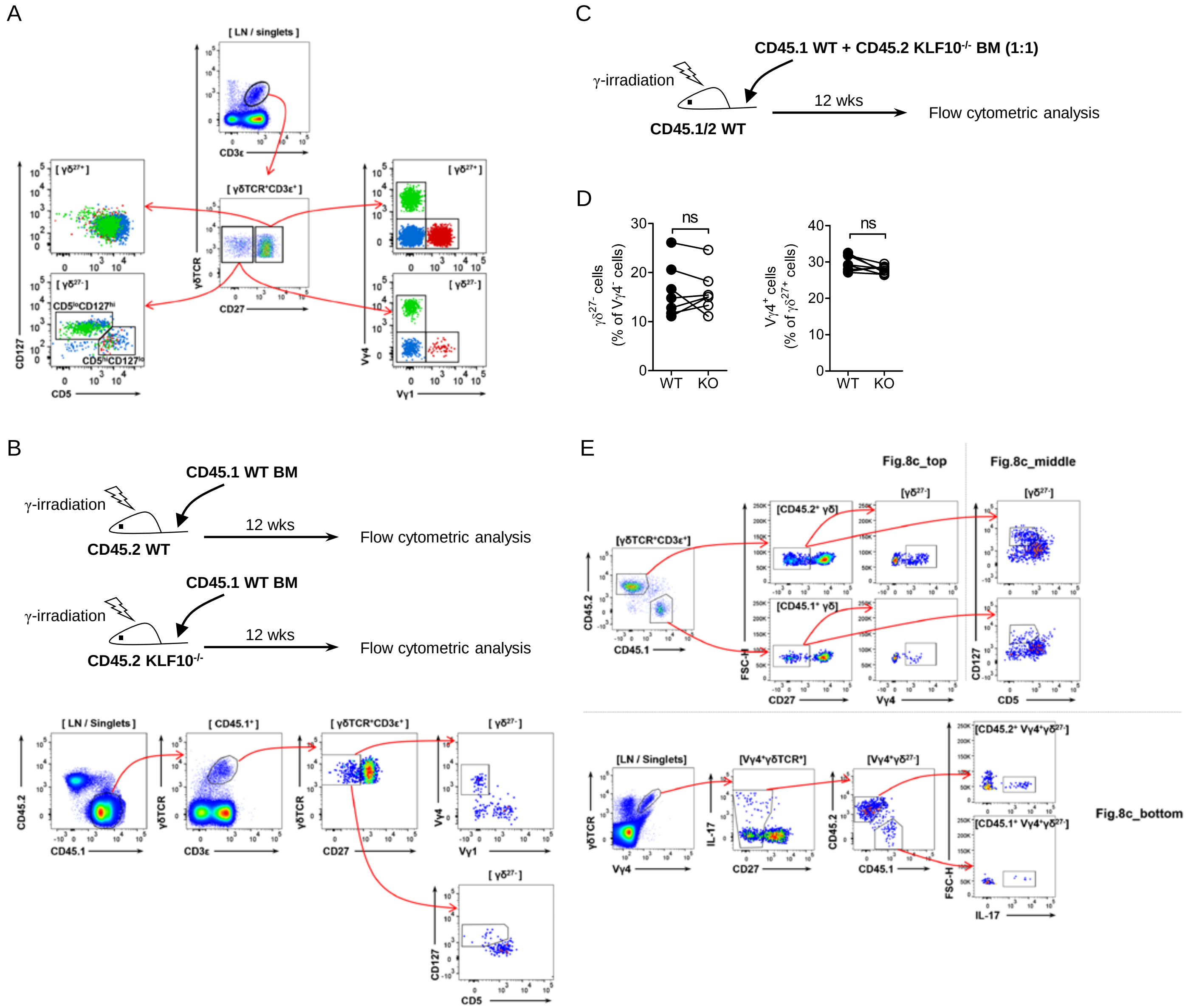

Supplementary Figure 6. The gating strategy of flow cytometric analysis and the scheme of bone marrow (BM) chimera experiments. (**A**) The gating strategy commonly used for defining γδ subsets, based on CD3ε, γδTCR, CD27, Vγ1, Vγ4, CD5 and CD127 antigens. (**B**) The experimental scheme and gating strategy used in **Fig. 8A**. (**C**) The experimental scheme for the mixed BM chimera setting used in **Fig. 8B** and **C**. (**D**) The percent of $\gamma\delta^{27-}$ cells among WT and KO $V\gamma4^-$ cells or, $V\gamma4^-$ cells among WT and KO $\gamma\delta^{27+}$ cells in the mixed BM chimera setting. (**E**) The gating strategy used in **Fig. 8C**.
